# Tomato TFT1 Is Required for PAMP-Triggered Immunity and Mutations that Prevent T3S Effector XopN from Binding to TFT1 Attenuate *Xanthomonas* Virulence

**DOI:** 10.1371/journal.ppat.1002768

**Published:** 2012-06-14

**Authors:** Kyle W. Taylor, Jung-Gun Kim, Xue B. Su, Chris D. Aakre, Julie A. Roden, Christopher M. Adams, Mary Beth Mudgett

**Affiliations:** 1 Department of Biology, Stanford University, Stanford, California, United States of America; 2 Stanford Mass Spectrometry, Stanford University, Stanford, California, United States of America; Michigan State University, United States of America

## Abstract

XopN is a type III effector protein from *Xanthomonas campestris* pathovar *vesicatoria* that suppresses PAMP-triggered immunity (PTI) in tomato. Previous work reported that XopN interacts with the tomato 14-3-3 isoform TFT1; however, TFT1's role in PTI and/or XopN virulence was not determined. Here we show that TFT1 functions in PTI and is a XopN virulence target. Virus-induced gene silencing of *TFT1* mRNA in tomato leaves resulted in increased growth of Xcv *ΔxopN* and Xcv *ΔhrpF* demonstrating that TFT1 is required to inhibit Xcv multiplication. *TFT1* expression was required for Xcv-induced accumulation of *PTI5*, *GRAS4*, *WRKY28*, and *LRR22* mRNAs, four PTI marker genes in tomato. Deletion analysis revealed that the XopN C-terminal domain (amino acids 344–733) is sufficient to bind TFT1. Removal of amino acids 605–733 disrupts XopN binding to TFT1 in plant extracts and inhibits XopN-dependent virulence in tomato, demonstrating that these residues are necessary for the XopN/TFT1 interaction. Phos-tag gel analysis and mass spectrometry showed that XopN is phosphorylated in plant extracts at serine 688 in a putative 14-3-3 recognition motif. Mutation of S688 reduced XopN's phosphorylation state but was not sufficient to inhibit binding to TFT1 or reduce XopN virulence. Mutation of S688 and two leucines (L64,L65) in XopN, however, eliminated XopN binding to TFT1 in plant extracts and XopN virulence. L64 and L65 are required for XopN to bind TARK1, a tomato atypical receptor kinase required for PTI. This suggested that TFT1 binding to XopN's C-terminal domain might be stabilized via TARK1/XopN interaction. Pull-down and BiFC analyses show that XopN promotes TARK1/TFT1 complex formation *in vitro* and *in planta* by functioning as a molecular scaffold. This is the first report showing that a type III effector targets a host 14-3-3 involved in PTI to promote bacterial pathogenesis.

## Author Summary

Bacterial pathogens of plants and animals employ the type III secretion system to secrete and translocate effector proteins into host cells to suppress defense responses. Biochemical analyses have revealed that several effector proteins mimic host enzyme activities to directly interfere with pathogen perception, defense signal transduction, and/or secretion of antimicrobial compounds. The XopN effector from *Xanthomonas campestris* pathovar *vesicatoria*, the causal agent of bacterial spot on tomato and pepper plants, suppresses PAMP-triggered immunity (PTI) but structural modeling predicts it encodes a unique scaffolding-like protein with no obvious enzymatic fold. We postulate that XopN mediates its virulence function by associating with host proteins regulating defense. XopN was previously shown to physically interact with the tomato 14-3-3 protein TFT1 *in planta*. The significance of TFT1 in plant immune signaling and the relevance of the XopN/TFT1 interaction were not determined. Here we show that TFT1 is a positive regulator of PTI in tomato required to inhibit Xcv growth. Moreover, we provide evidence that TFT1 is a bona fide target of XopN because mutations that disrupt XopN/TFT1 binding also eliminate XopN-dependent virulence in tomato. This is the first example of a bacterial effector targeting a 14-3-3 associated with host immunity.

## Introduction

Plant immunity to bacterial pathogens requires a complex detection and signaling network. Plants use pattern recognition receptors (PRRs) at the host cell surface to detect conserved microbial-associated molecular patterns also referred to as pathogen-associated molecular patterns (PAMPs) (*e.g.* flagellin and elongation factor EF-Tu) [1]. Activation of PRRs initiates downstream signaling events that lead to the production of reactive oxygen species, stimulation of mitogen-activated protein kinase (MAPK) cascades, defense gene induction, and callose deposition at the plant cell wall [2]–[6]. These host responses are sufficient to limit the growth of a broad range of potential pathogens and are collectively referred to as PAMP-triggered immunity or PTI [7].

In response, phytopathogenic bacteria evolved the type III secretion (T3S) to combat this layer of plant immunity [8]–[11]. T3S systems are widely conserved amongst bacteria and in most cases are critical virulence determinants [12], [13]. The T3S apparatus mediates the secretion and translocation of effector proteins from the pathogen into eukaryotic host cells. Progress in understanding T3S effector function in plants has revealed that several proteins encode enzymes with different activities (*e.g.* SUMO protease, cysteine proteases, protein tyrosine phosphatase, E3 ligase, ADP-ribosyltransferase, phosphothreonine lyase and acetyltransferase) [14]–[21]. Some of these enzymes have been shown to target critical signaling components of the plant defense machinery [17], [22]–[27]. In response, plants have evolved another layer of immune signaling known as effector-triggered immunity (ETI), which employs the use of resistance (R) proteins to monitor and amplify defense signal transduction to limit pathogen invasion and spread [7]. Plant immune signaling is thus complex and multilayered. Defining the mechanism(s) by which T3S effectors suppress PTI and/or ETI is fundamental to understand and combat bacterial pathogenesis.

We are interested in elucidating how T3S effectors with no apparent enzymatic function perturb plant defense signal transduction. The XopN effector [28] from *Xanthomonas campestris* pathovar *vesicatoria* (Xcv), the causal agent of bacterial spot disease of tomato and pepper [29], is a virulence factor that is widely conserved in *Xanthomonas* spp. [30], [31]. XopN suppresses PTI at early stages of infection in tomato and *Arabidopsis* by unknown mechanism(s) [28]. Structural modeling predicts that XopN contains anti-parallel, α-helical tandem repeats [30]. Proteins with such structural features resemble scaffolds or adapters that coordinate protein-protein interactions [32], [33]. We therefore hypothesize that XopN mediates its virulence function inside plant cells by interfering with host protein activity and/or the organization of signaling cascades by physical association. Consistent with this hypothesis, XopN localizes at the host plasma membrane-cytoplasmic interface and strongly interacts with the kinase domain of the tomato atypical receptor kinase1 (TARK1) [28]. TARK1 encodes a membrane protein with five extracellular leucine rich repeats and a non-RD cytoplasmic kinase domain [34]. Although TARK1 does not possess any apparent kinase activity, it is required to inhibit Xcv growth, indicating that it plays a role in PTI in tomato [28].

In addition to TARK1, XopN also interacts with four tomato 14-3-3 isoforms – TFT1, TFT3, TFT5 and TFT6 [28]. The interaction between XopN and TFT1 was confirmed because XopN binding to TFT1 in yeast was the strongest [28], and TFT1 mRNA levels were known to accumulate in response to pathogen infection in tomato [35]. XopN was shown to bind TFT1 in *Nicotiana benthamiana* by bimolecular fluorescence complementation and pull-down analysis [28]. While TFT1 was localized throughout the plant cytoplasm and nucleus, the XopN/TFT1 interaction was observed only in the plant cytoplasm in close association with the plasma membrane, reflecting XopN's localization pattern [28]. The relevance of the XopN/TFT1 binding during Xcv infection in tomato, however, was not further investigated.

TFT1 is a member of a large 14-3-3 family in tomato consisting of 12 isoforms [35], [36]. 14-3-3s are acidic, phosphopeptide-binding proteins that are ubiquitously found among eukaryotes. 14-3-3s bind a diverse set of client proteins involved in distinct cellular processes (*e.g.* primary metabolism, signal transduction, transcription, protein trafficking, cell cycle, development, apoptosis, and stress responses), revealing that 14-3-3s are critical regulators of protein function [37]–[39]. Although 14-3-3s have no intrinsic enzymatic activity, they are known to regulate client activity by three principal mechanisms – clamping, masking, and scaffolding [37]. These mechanisms directly influence client stability, conformation, trafficking, and/or protein-protein interactions.

In terms of plant immunity, it has been known for some time that 14-3-3 mRNA levels are up-regulated in plants in response to pathogen attack [36]. The precise role of 14-3-3s in the regulation of plant defense signal transduction is not clear. Two recent biochemical studies indicate that 14-3-3s interact with central components of the plant defense machinery to positively regulate immunity. The *Arabidopsis* 14-3-3 isoform λ (GF14λ) binds to the atypical R protein RPW8.2 [40] and is implicated in the regulation of programmed cell death (PCD) and resistance to powdery mildew [40]. The tomato 14-3-3 isoform 7 (TFT7) interacts with two mitogen-activated protein kinases, MAPKKKα and MKK2, that function in multiple R protein signaling pathways revealing that this 14-3-3 coordinates the activity and possibly the assembly of multiple defense components [41].

Interestingly, host 14-3-3s have been shown to specifically interact with T3S effectors from animal and plant bacterial pathogens [28], [42]–[44]. In the case of exoenzyme S (ExoS) from *Pseudomonas aeruginosa*, 14-3-3 binding is required for activation of ExoS-dependent ADP-ribosyltransferase activity [45] and cell death in the mouse model of pneumonia [46]. Host 14-3-3s are thus recruited during infection to promote ExoS virulence. Evidence showing that T3S effectors directly antagonize 14-3-3 function(s) to inhibit defense signaling has been lacking, although prime candidates include XopN from Xcv and HopM1 from *Pseudomonas syringae*. Both effectors are known to bind specific plant 14-3-3 isoforms and suppress PTI [28], [44].

In this study, we characterized the role of the tomato 14-3-3 TFT1 in PTI and examined the biological relevance of the XopN/TFT1 interaction during Xcv infection in tomato. We performed virus-induced gene silencing (VIGS) in tomato to determine if TFT1 is required to inhibit Xcv growth. In addition, we performed structure-function analyses to identify the domains and amino acids in XopN that are required for TFT1 binding and XopN-dependent virulence in tomato. Our data indicate that TFT1 is a positive regulator of PTI in tomato required to inhibit Xcv pathogenesis and is a virulence target of the XopN effector.

## Results

### TFT1 mRNA levels increase in response to Xcv

**Figure 1:**
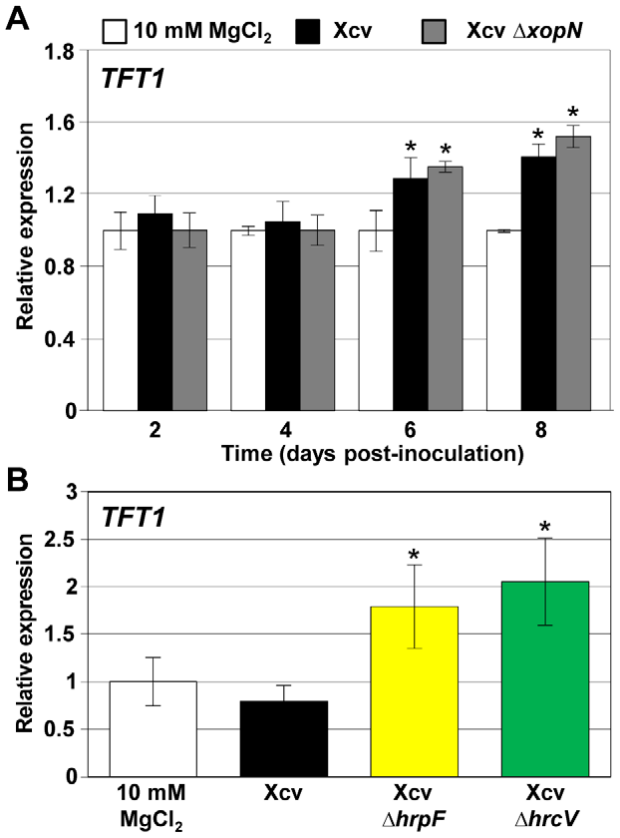
*TFT1* is a PTI-induced gene in tomato. (**A**) Susceptible VF36 tomato leaves were inoculated with 10 mM MgCl_2_ (white bars), 1×10^5^ CFU/mL of Xcv (black bars), or Xcv *ΔxopN* (grey bars). (**B**) Susceptible VF36 tomato leaves were inoculated with 10 mM MgCl_2_ (white bars), 2×10^8^ CFU/mL of Xcv (black bars), Xcv *ΔhrpF* (yellow bars), or Xcv *ΔhrcV* (green bars). Total RNA was isolated at 2, 4, 6, and 8 DPI (**A**) or 6 HPI (**B**). Q-PCR was performed to monitor *TFT1* mRNA levels. *Actin* expression was used to normalize the expression value in each sample, and relative expression values were determined against the average value of the sample infiltrated with 10 mM MgCl_2_ at each time point. Error bars indicate SD for three (**A**) and four (**B**) plants. Asterisk indicates significant difference (*t* test, P<0.05) relative to the 10 mM MgCl_2_ control at 6 or 8 DPI (**A**) or 6 HPI (**B**).

To determine if Xcv infection alters *TFT1* mRNA abundance, we monitored *TFT1* mRNA levels in 4-week old VF36 tomato leaflets inoculated with 10 mM MgCl_2_ or a 10 mM MgCl_2_ suspension containing a low titer (1×10^5^ colony forming units (CFU)/mL) of Xcv or Xcv *ΔxopN*. These conditions mirror those used in bacterial growth curves to monitor changes in pathogen multiplication and disease symptom development over a two-week period. Quantitative real-time RT-PCR (Q-PCR) revealed that the relative level of *TFT1* mRNA was similar in all leaf samples at 2 and 4 days post-inoculation (DPI). At 6 and 8 DPI, *TFT1* mRNA levels significantly increased in both the Xcv- and Xcv *ΔxopN*-infected leaf tissues (Figure 1A). This is the time period when Xcv and Xcv *ΔxopN* titers begin to differ significantly within the leaf tissue due to the impact of PTI in tomato [28]. It is also the time point when XopN-dependent suppression of PTI marker genes occurs [28]. These data indicate that Xcv infection induces *TFT1* mRNA levels tomato leaves in a XopN-independent manner.

We next determined if *TFT1* is a PAMP-induced gene. Tomato leaflets were inoculated with 10 mM MgCl_2_ or a 10 mM MgCl_2_ suspension containing a high titer (2×10^8^ CFU/mL) of Xcv, Xcv *ΔhrpF*, or Xcv *ΔhrcV*. The *hrpF* mutant lacks the putative T3S translocon HrpF required for effector delivery into plant cells [47]. The *hrcV* mutant lacks a structural membrane component of the T3S apparatus required for effector secretion from the pathogen [48]. Host responses to Xcv *ΔhrcV* represent the recognition of a collection of Xcv PAMPs that are not suppressed by Xcv T3S effectors. *A* high titer and short time course was used to ensure similar bacterial cell number within the leaves at the indicated time points because PTI inhibits Xcv *ΔhrpF* and Xcv *ΔhrcV* growth much more than wild type Xcv. Q-PCR was used to determine TFT1 mRNA abundance in the inoculated leaves at 6 hours post-inoculation (HPI). *TFT1* mRNA levels were significantly higher in tomato leaves inoculated with Xcv *ΔhrpF* or Xcv *ΔhrcV* compared with those infected with Xcv or 10 mM MgCl_2_ (Figure 1B), revealing that *TFT1* is a PAMP-induced gene that is regulated in a T3S-dependent manner. Elevated *TFT1* mRNA levels in Xcv *ΔhrpF*-infected leaves in the high dose experiment (Figure 1B) are consistent with our findings in the low dose experiments (Figure 1A). The early induction of *TFT1* mRNA levels in response to a high dose of *Xcv ΔhrcV* suggests that TFT1 plays a role in PTI and qualifies it as a PTI marker gene in tomato.

### *TFT1*-silenced tomatoes are more susceptible to infection with the Xcv *ΔhrpF* mutant

**Figure 2:**
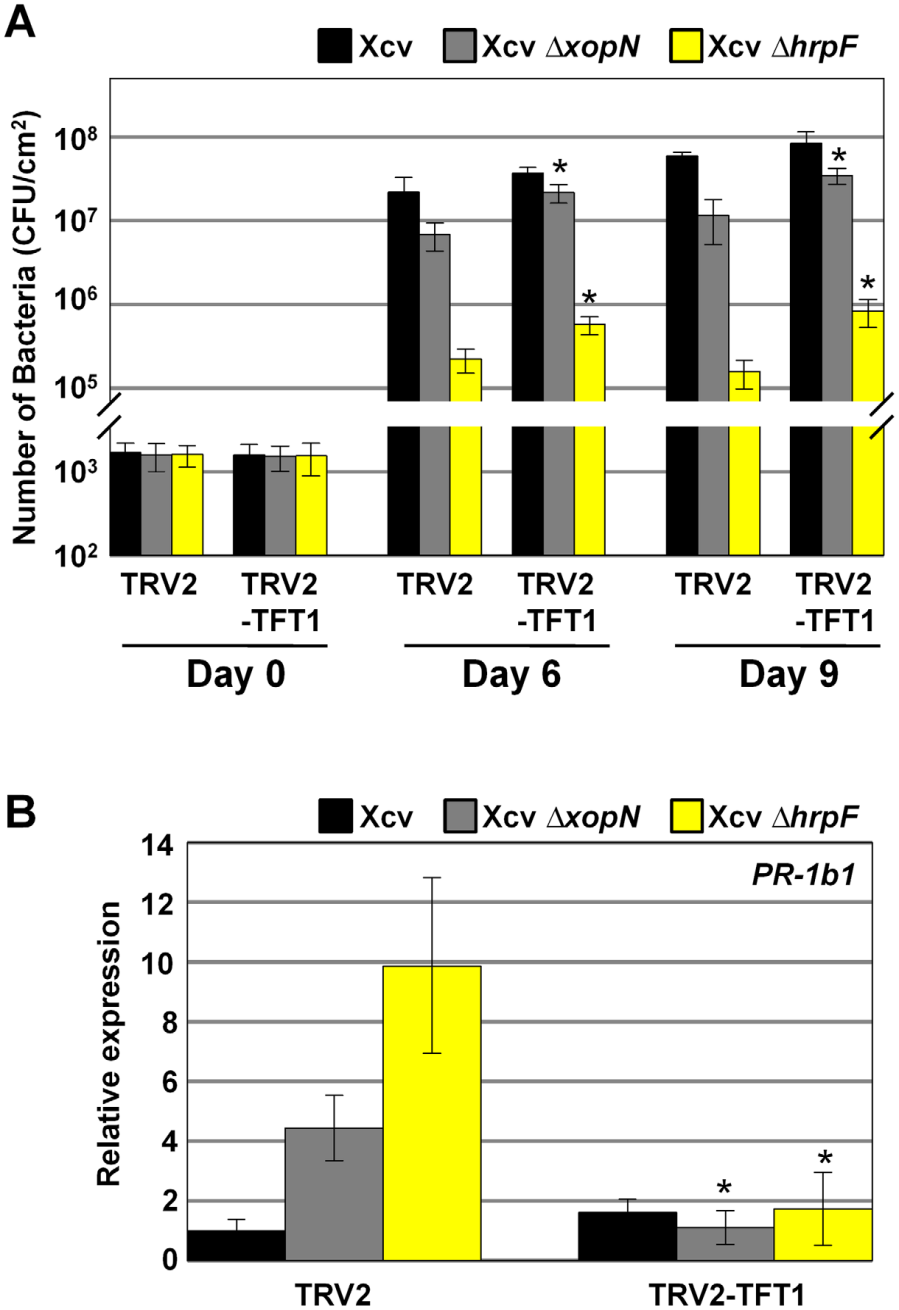
Reduced *TFT1* mRNA expression in VIGS tomato leaves promotes Xcv *ΔhrpF* and Xcv *ΔxopN* growth. (**A**) Growth of Xcv, Xcv *ΔhrpF*, or Xcv *ΔxopN* in control (TRV2) and *TFT1*-silenced (TRV2-TFT1) susceptible VF36 tomato lines. Leaves were inoculated with 1×10^5^ CFU/mL of pathogen. Bacterial growth was quantified at 0, 6, and 9 DPI. Data points represent mean CFU/cm^2^ ± SD of four plants. Asterisk indicates significant difference (*t* test, P<0.05) in the infected TRV2-TFT1 lines compared to the similarly infected TRV2 lines. (**B**) Relative *PR-1b1* mRNA levels in 4 control (TRV2) and 4 *TFT1*-silenced (TRV2-TFT1) tomato lines. Total RNA isolated from infected leaves in (**A**) on day 6 was used for Q-PCR. *Actin* mRNA expression was used to normalize the expression value in each sample. Error bars indicate SD for four plants. Asterisk indicates significant difference (*t* test, P<0.05) in the infected TRV2-TFT1 lines compared to the similarly infected TRV2 lines.

Considering that Xcv *ΔhrpF* inoculation significantly increases the level of *TFT1* mRNA (Figure 1B), we determined whether or not *TFT1* expression in tomato leaves was required to inhibit the growth of Xcv *ΔhrpF*. We performed transient virus-induced gene silencing (VIGS) using a tobacco rattle virus (TRV)-based system to reduce *TFT1* mRNA levels in susceptible VF36 tomato leaves. Bacterial growth curves were performed using the vector control and *TFT1*-silenced plants to determine if Xcv *ΔhrpF* growth increases when *TFT1* expression is reduced. Leaflets from the same fully expanded branch were inoculated with a 1×10^5^ CFU/mL suspension of wild type Xcv or the Xcv *ΔhrpF* mutant. The number of bacteria in each leaflet was quantified at 0, 6, and 9 DPI (Figure 2A). Total RNA was isolated from the same leaflets at 0 DPI to measure *TFT1* mRNA levels by Q-PCR (Figure S1A). We also measured the mRNA levels of *TFT3* and *TFT6* to verify specificity for the VIGS construct (Figure S1B,C). These isoforms were chosen because they are highly homologous to *TFT1* at the nucleotide level and they were shown to only weakly interact with XopN [28].

Relative to the vector control plants (*i.e.* TRV2 lines), *TFT1* mRNA levels were reduced approximately 4-fold in leaflets from four independent *TFT1*-silenced tomato plants (*i.e.* TRV2-TFT1 lines, Figure S1A). Reduced *TFT1* mRNA levels in the TRV2-TFT1 leaves correlated with a significant increase in growth of Xcv *ΔhrpF* at 6 and 9 DPI relative to the growth of Xcv *ΔhrpF* detected in the TRV2 vector control leaves (Figure 2A). Growth of wild type Xcv was not significantly different in the TRV2-TFT1 leaves compared to the TRV2 vector control leaves (Figure 2A). Taken together, our data indicate that TFT1 is required to inhibit Xcv *ΔhrpF* multiplication in tomato, further substantiating a role for this 14-3-3 in PTI.

### Silencing *TFT1* mRNA expression partially restores Xcv *ΔxopN* growth

We hypothesized that XopN may directly bind to TFT1 to suppress PTI during Xcv infection. If XopN binding to TFT1 is critical for XopN-dependent virulence, then reduced TFT1 levels *in planta* should increase and/or fully restore the growth of the Xcv *ΔxopN* null mutant [28] in tomato leaves. To test this, we quantified Xcv *ΔxopN* growth in the same set of control and *TFT1*-silenced tomato plants described above (Figure 2A). In the TRV2 vector control line containing *TFT1* mRNA (Figure S1A), the titer of Xcv was 5-fold higher than that of Xcv *ΔxopN* at 9 DPI (Figure 2A), consistent with the levels typically quantified in wild type VF36 tomato plants [28]. By contrast, in the *TFT1*-silenced lines with significantly reduced levels of *TFT1* mRNA (Figure S1A), the titer of Xcv in the TRV2-TFT1 leaflets was only 2.4-fold higher than that of the Xcv *ΔxopN* in the TRV2-TFT1 leaflets (Figure 2A). These data indicate that increased growth of the Xcv *ΔxopN* mutant correlates with reduced *TFT1* mRNA expression in tomato. This phenotype was observed in three independent *TFT1* VIGS experiments (data not shown). It is noteworthy to mention that symptom development was not dramatically affected in the *TFT1*-silenced lines. That is, Xcv-infected leaves generally collapsed before the Xcv *ΔxopN*-infected leaves, a phenotype typically observed in wild type tomato plants [28]. Only slight differences in the onset and severity of tissue chlorosis and necrosis were observed in some *TFT1* VIGS lines depending the severity of *TFT1* silencing.

We also monitored the mRNA levels of *PR-1b1*, a tomato pathogenesis-related gene, in the same VIGS lines to determine if *TFT1* expression is required for maximal *PR-1b1* gene induction during Xcv *ΔxopN* infection. We previously showed that XopN is required to suppress *PR-1b1* mRNA levels in tomato leaves during Xcv infection [28]. As expected, *PR-1b1* mRNA levels (Figure 2B) increased at 6 DPI in TRV2 vector control leaflets infected with Xcv *ΔxopN* or Xcv *ΔhrpF*. The levels of *PR-1b1* mRNA were however significantly lower in the TRV2-TFT1 leaflets infected with Xcv Δ*xopN* and Xcv *ΔhrpF* (Figure 2B). These data indicate that *TFT1* mRNA expression is required for maximal expression of this defense marker gene during Xcv infection in tomato.

### *TFT1* expression is required for Xcv-induced PTI mRNA accumulation

**Figure 3:**
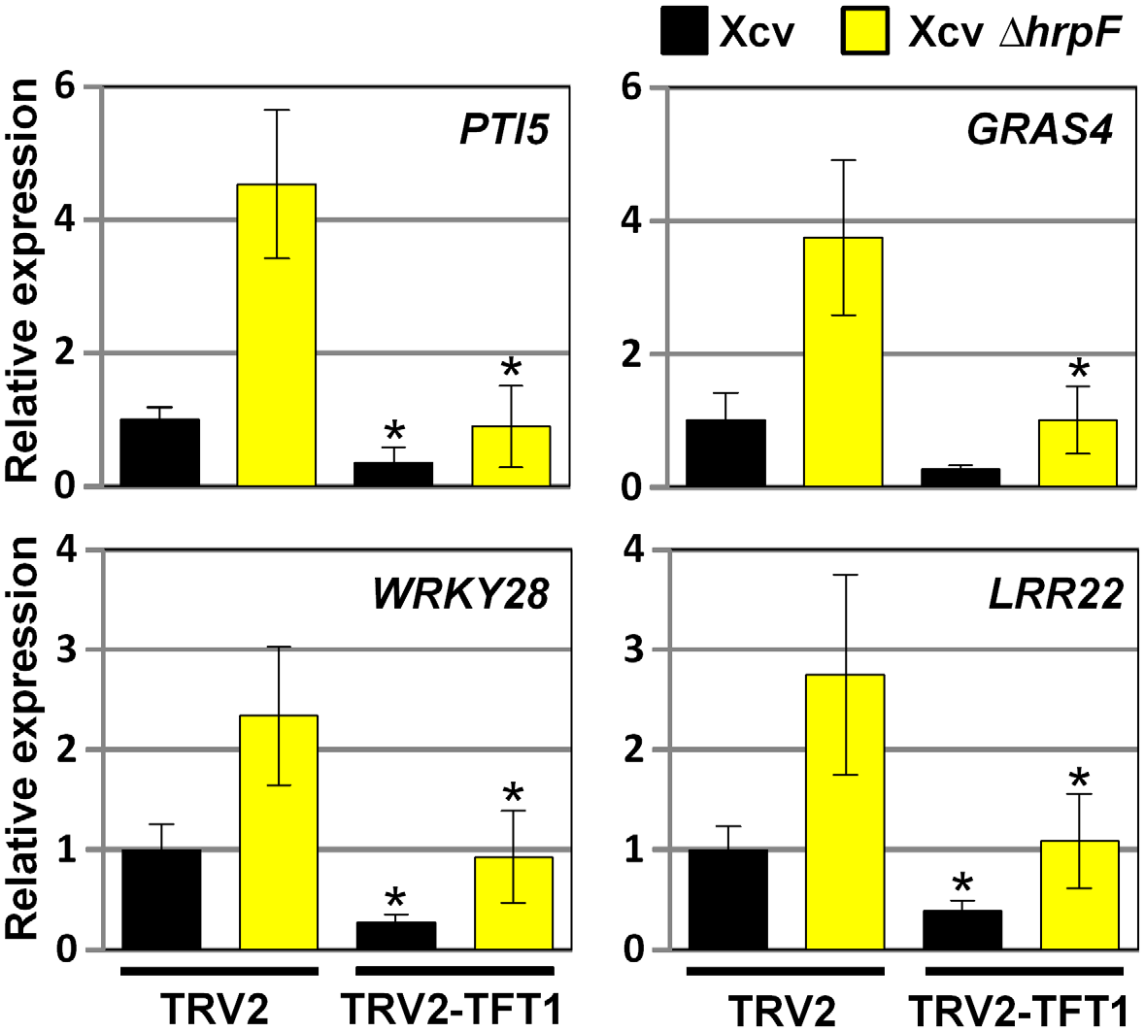
Reduced *TFT1* mRNA expression in VIGS tomato leaves correlates with reduced PTI marker mRNA abundance in response to Xcv infection. Relative mRNA levels for four PTI marker genes (*PTI5*, *GRAS4*, *WRKY28*, and *LRR22*) in 4 control (TRV2) and 4 *TFT1*-silenced (TRV2-TFT1) tomato lines. Leaflets on the same branch were inoculated with 1×10^5^ CFU/mL of Xcv or Xcv *ΔhrpF*. Total RNA isolated from inoculated leaves at 6 HPI was used for Q-PCR. *Actin* mRNA expression was used to normalize the expression value in each sample. Error bars indicate SD for four plants. Asterisk indicates significant difference (*t* test, P<0.05) in the infected TRV2-TFT1 lines compared to the similarly infected TRV2 lines.

Reduced *PR-1b1* mRNA levels in *TFT1*-silenced tomato leaves suggested that TFT1 may be required for other PTI marker gene expression in tomato. To examine this, we generated an independent set of control and *TFT1*-silenced tomato plants using VIGS. The plants were inoculated with a high titer (2×10^8^ CFU/mL) of Xcv or Xcv *ΔhrpF*. Total RNA was isolated at 6 HPI and then the level of four mRNAs (*i.e. PTI5*, *GRAS4*, *WRKY28*, and *LRR22*) known to be associated with PTI in tomato [28], [49] was quantified by Q-PCR. *TFT1* mRNA levels were significantly reduced (∼4-fold lower) in the TRV2-TFT1 leaves relative to the TRV2 control leaves (Figure S2). The mRNA levels for *PTI5*, *GRAS4*, *WRKY28*, and *LRR22* were significantly higher in TRV2 control leaves inoculated with Xcv *ΔhrpF* compared to those inoculated with Xcv (Figure 3). This indicates that Xcv suppresses the accumulation of these PTI marker mRNAs in a type III-dependent manner. *PTI5*, *GRAS4*, *WRKY28*, and *LRR22* mRNA levels in the TRV2-TFT1 leaves inoculated with Xcv *ΔhrpF* were significantly lower than those in the TRV2 leaves inoculated with Xcv *ΔhrpF* (Figure 3). *PTI5*, *WRKY28*, and *LRR22* mRNA levels were also significantly reduced in the Xcv-infected TRV2-TFT1 leaves relative to the Xcv-infected control leaves. Taken together, these data show that *TFT1* expression is required for the mRNA accumulation of these PTI marker genes in susceptible tomato leaves challenged with Xcv or Xcv *ΔhrpF*.

### The C-terminus of XopN is required for TFT1 binding

**Figure 4:**
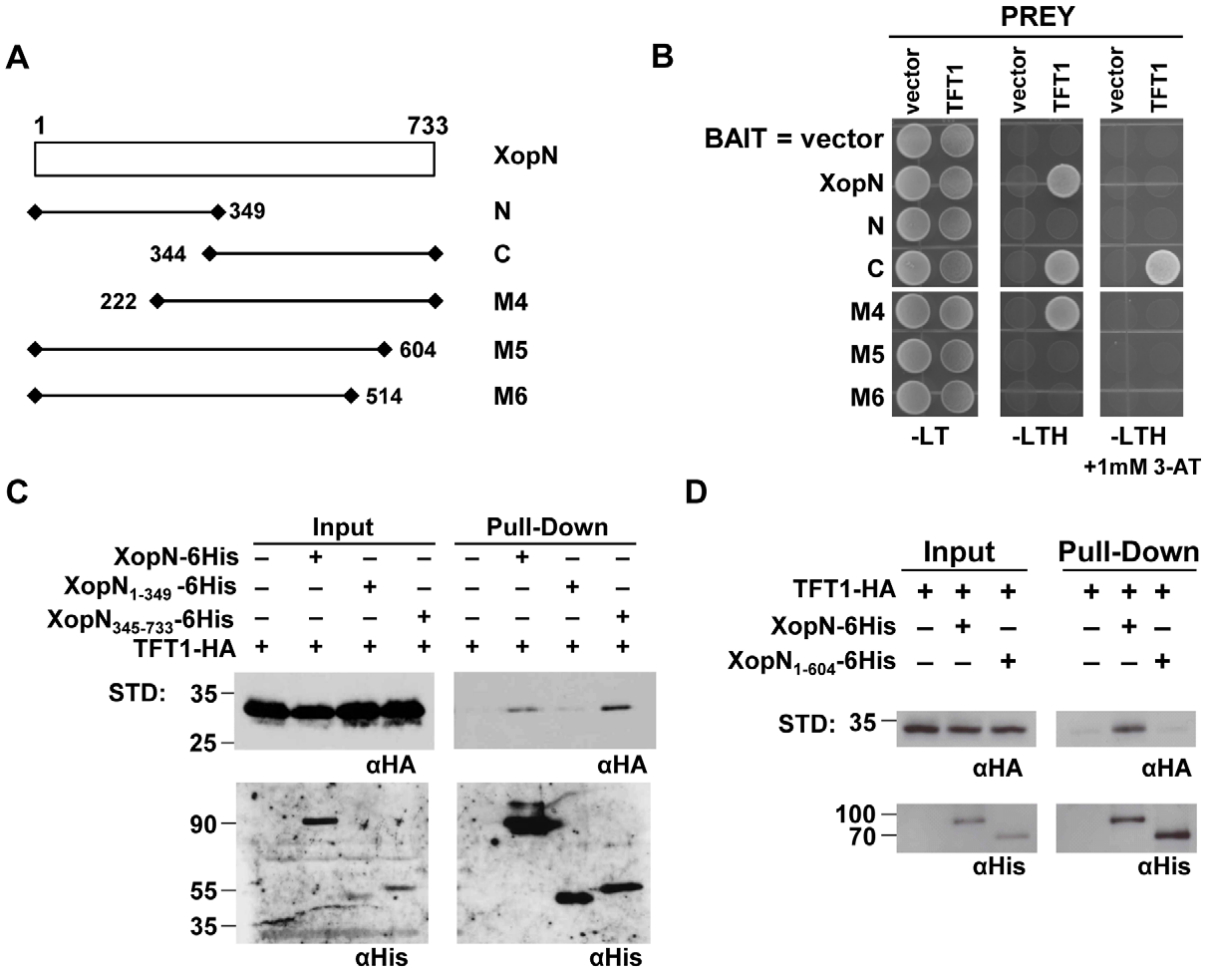
TFT1 associates with the C-terminal domain of XopN. (**A**) Schematic of XopN protein and various deletion mutants. Wild type and mutant *xopN* were cloned into pXDGATcy86(GAL4-DNA binding domain) to create DBD-XopN fusion proteins: XopN, XopN 1–733; N, 1–349; C, 344–733; M4, 222–733; M5, 1–604; and M6, 1–514. Numbering refers to amino acid residues in wild type XopN (733 amino acids). (**B**) TFT1 interaction with XopN mutant proteins in yeast. Yeast strain AH109 carrying pXDGATcy86 containing vector, XopN, N, C, M3, M4, M5 and M6 were independently transformed with the following PREY constructs: pGADT7(GAL4 activation domain) alone (Vector) or pGADT7 containing TFT1. Strains were spotted on nonselective (SD-LT) and selective (SD-LTH) media ± 1 mM 3-amino-1,2,4-triazole and then incubated at 30°C for 3d. (**C**) Pull-down analysis of TFT1-HA and XopN-6His, XopN_1–349_-6His, or XopN_345–733_-6His in *N. benthamiana* extracts. *N. benthamiana* leaves were inoculated with a suspension of 6×10^8^ CFU/mL of *A. tumefaciens* co-expressing TFT1-HA and XopN-6His, XopN_1–349_-6His, or XopN_345–733_-6His. After 48 h, protein was extracted, purified by Ni^+^ affinity chromatography, and then analyzed by protein gel blot analysis using anti-His and anti-HA sera. Expected protein MW: TFT1-HA = 29.3 kDa; XopN-6His = 78.7 kDa; XopN_1–349_-6His = 38.0 kDa; XopN_345–733_-6His = 42.0 kDa; +, protein expressed; −, vector control. STD, molecular weight standard. (**D**) Pull-down analysis of TFT1-HA and XopN-6His or XopN_1–604_-6His in *N. benthamiana* extracts. *N. benthamiana* leaves were hand-inoculated with a mixed suspension of 1×10^8^ CFU/mL of *A. tumefaciens* expressing TFT1-HA and 4×10^8^ CFU/mL of XopN-6His or XopN_1–604_-6His. Samples were processed as described in (**C**). Expected protein MW: TFT1-HA = 29.3 kDa; XopN-6His = 78.7 kDa; XopN_1–604_-6His = 64.9 kDa; +, protein expressed; −, vector control. STD, molecular weight standard.

If TFT1 is a bona fide virulence target for XopN, then mutations that disrupt XopN/TFT1 binding should attenuate Xcv virulence in tomato. To test this, we roughly mapped the TFT1 binding site in XopN. A series of N- and C-terminal XopN deletion mutants were constructed and then assayed for interaction with TFT1 using the GAL4-based two-hybrid assay in yeast. Wild type XopN and five XopN deletion mutants (Figure 4A) were cloned into pXDGATcy86 containing the GAL4 DNA-binding domain (DBD) to create DBD-XopN fusion proteins (*i.e.* XopN, N, C, M4, M5 and M6). TFT1 was cloned into pGADT7 containing the GAL4 activation domain (AD) to create AD-TFT1. The DBD-XopN fusion proteins (BAIT) were then tested for interaction with AD-TFT1 (PREY) in targeted yeast two-hybrid assays. Protein expression levels in yeast are shown in Figure S3. The XopN derivatives M4, M5, and M6 were highly expressed. Derivative C expression was similar to XopN, whereas derivative N expression was less than XopN. TFT1 interacted with the C-terminal (C) but not the N-terminal (N) domain of XopN (Figure 4B). Selection on media containing 1 mM 3-amino-1,2,4-triazole, a competitive inhibitor of the *HIS3* gene product, indicated that TFT1 binding to the C-terminal domain is stronger than binding to full-length XopN (Figure 4B). TFT1 also interacted with the M4 mutant lacking XopN amino acids 1–221 (Figure 4B) but failed to interact with the M5 and M6 mutants lacking the C-terminal 605–733 and 515–733 amino acids of XopN, respectively (Figure 4B). These data suggest that TFT1 binds preferentially to XopN's C-terminal domain in yeast.

We next verified that TFT1 binds to the C-terminal domain of XopN *in planta* by performing a Ni-NTA affinity pull-down assay using *N. benthamiana* protein extracts. *N. benthamiana* leaves were hand-infiltrated with a 6×10^8^ CFU/mL suspension of *A. tumefaciens* expressing TFT1-HA alone or coexpressing TFT1-HA and XopN-6His (full-length), XopN_1–349_-6His (N-terminal domain), or XopN_345–733_-6His (C-terminal domain). Leaf samples were collected at 48 HPI and total soluble protein extracts were isolated. Using Ni-NTA agarose beads, XopN-6His, XopN_1–349_-6His or XopN_345–733_-6His were purified by affinity chromatography from the protein extracts and then analyzed by protein gel blot analysis. As expected, TFT1-HA copurified with XopN-6His (Figure 4C). More TFT1-HA copurified with XopN_345–733_-6His than XopN-6His consistent with the finding that TFT1 exhibited stronger binding to the C-terminal domain of XopN than the full-length polypeptide in the yeast two-hybrid assay. Only a low level of TFT1-HA was detected in the pull-down with XopN_1–349_-6His (Figure 4C). Although XopN_1–349_-6His was less stable than XopN-6His and XopN_345–733_-6His in total protein extracts, the protein was highly enriched by affinity chromatography (Figure 4C) and able to copurify TARK1-HA in control experiments (Figure S4). TARK1 is an atypical receptor kinase that binds to the N-terminus of XopN [28]. These data suggest that the large C-terminal deletion of XopN did not grossly alter the structure of its N-terminal domain. The data also suggest that TFT1 binds preferentially to the C-terminus of XopN.

The importance of XopN's C-terminal domain for binding to TFT1 was further supported by pull-down analysis using the M5 mutant (amino acids 1–604) (Figure 4A). TFT1-HA and XopN-6His or XopN_1–604_-6His were transiently co-expressed in *N. benthamiana* and affinity purified as described above. Weak binding of TFT1-HA to the Ni-NTA agarose beads was observed. TFT1-HA was enriched in the XopN-6His pull-down but not in the XopN_1–604_-6His pull-down (Figure 4D). This indicates that some of the amino acid residues between 605–733 in XopN are required for TFT1 binding.

### XopN C-terminus is important for XopN-dependent virulence in tomato

**Figure 5:**
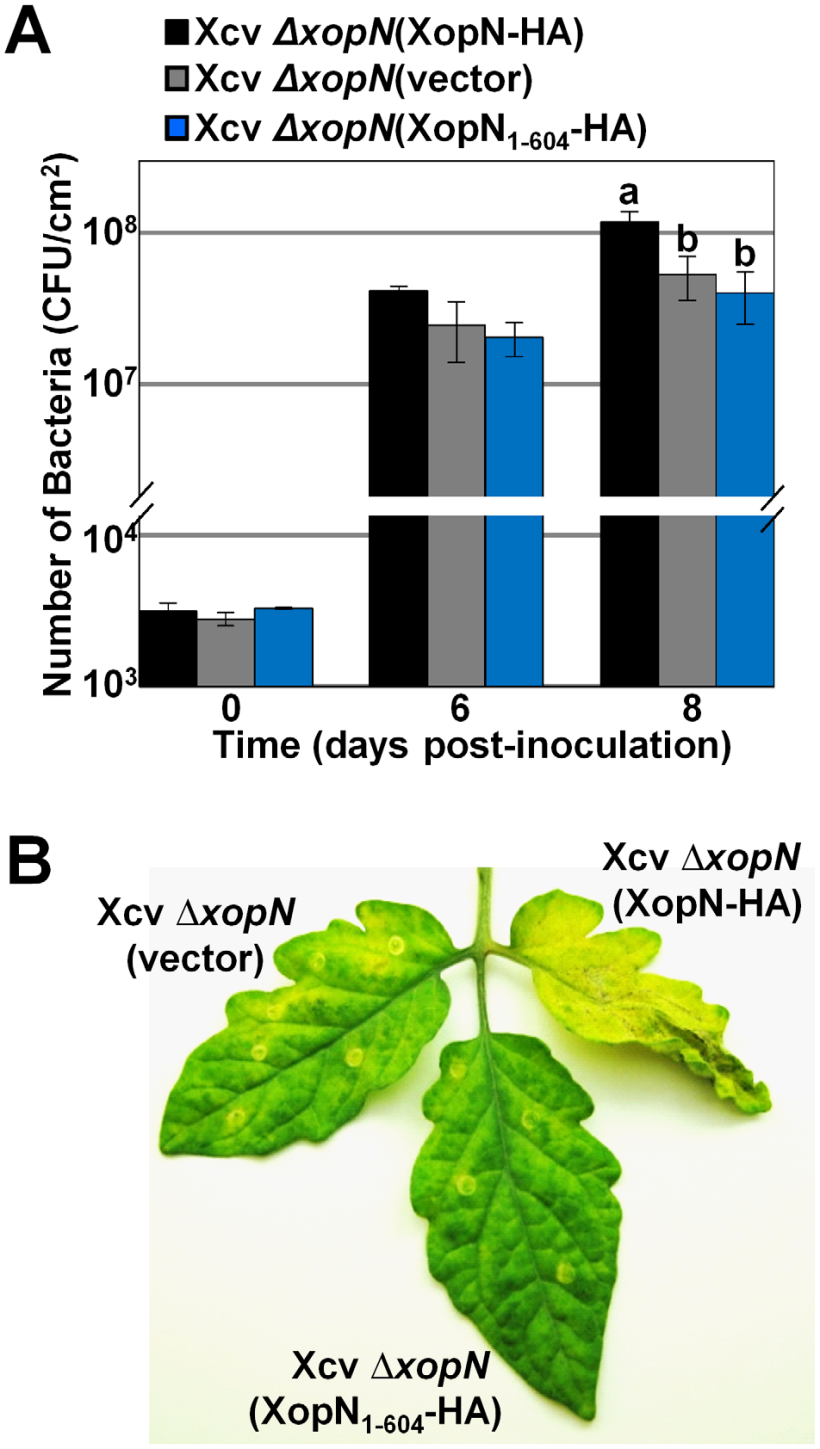
XopN residues 605–733 are important for TFT1 binding and contribute to XopN-dependent virulence in tomato. (**A**) Growth of Xcv Δ*xopN* (vector) (grey bars), Xcv Δ*xopN* (XopN_1–604_-HA) (blue bars), or Xcv Δ*xopN* (XopN-HA) (black bars) in susceptible tomato VF36 leaves. Leaves were inoculated with a 1×10^5^ CFU/mL suspension of bacteria. Number of bacteria in each leaf was quantified at 0, 6 and 8 DPI. Data points represent mean CFU/cm^2^ ± SD of three plants. Analysis was repeated at least three times. Vector = pVSP61. Different letters at day 8 indicate statistically significant (one-way analysis of variance and Tukey's HSD test, P<0.05) differences between the samples. (**B**) Phenotype of tomato leaves inoculated with strains described in (**A**). Leaves were photographed at 12 DPI. Similar phenotypes were observed in 3 independent experiments.

To determine the importance of the XopN's C-terminal residues for XopN-dependent virulence in tomato, we examined growth and symptom development of the Xcv *ΔxopN* mutant expressing wild type XopN-HA, the C-terminal deletion mutant XopN_1–604_-HA, or the vector control. All of the XopN constructs contained the *xopN* promoter (690 bp 5′ of the *xopN* ATG start site) that was shown to be sufficient to express the wild type XopN protein and restore full virulence of the Xcv *ΔxopN* mutant strain [28]. Susceptible VF36 tomato leaflets of 4 week-old plants were hand-infiltrated with a 1×10^5^ CFU/mL suspension of bacteria. At 0, 6, and 8 DPI, the number of bacteria in each leaflet was quantified. As expected, the titer of the Xcv *ΔxopN* (vector) strain was approximately 5-fold lower than that of the complemented Xcv *ΔxopN* (XopN-HA) strain demonstrating that XopN is required for maximal Xcv growth *in planta* (Figure 5A). Reduced Xcv *ΔxopN* (vector) growth resulted in reduced leaf symptom development (Figure 5B). Xcv *ΔxopN* expressing XopN_1–604_-HA exhibited reduced bacterial growth and symptom development comparable to that of the Xcv *ΔxopN* (vector) null mutant (Figure 5A,B). Protein gel blot analysis of total protein extracted from the Xcv strains confirmed that XopN_1–604_-HA protein levels were similar to that of XopN-HA (Figure S5A). Thus, XopN amino acid residues 605–733, which are critical for TFT1 binding, are also required for XopN-dependent virulence in tomato.

### XopN is phosphorylated in plant extracts

**Figure 6:**
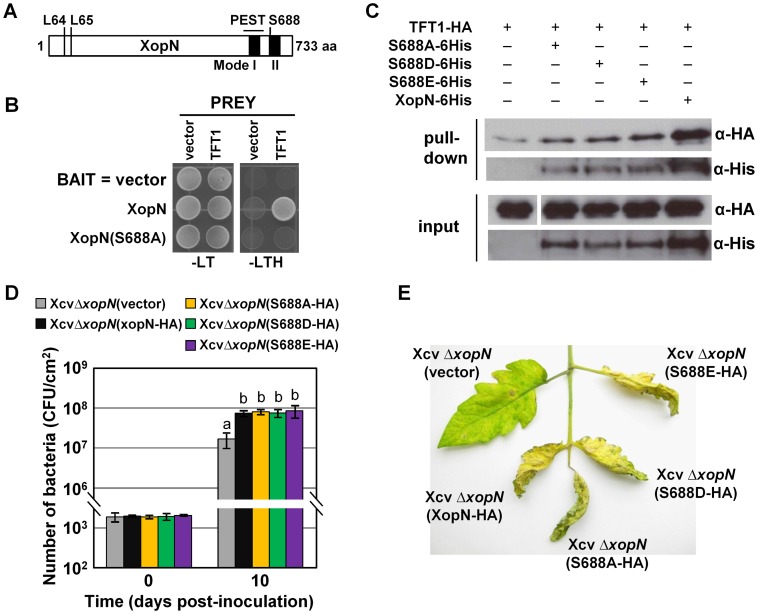
Serine 688 in XopN is required for TFT1 binding in yeast but not *in planta*. (**A**) Schematic of putative 14-3-3 motifs in XopN protein. Black boxes represent regions for putative Mode I and II 14-3-3 binding motifs. Mode II site contains S688. PEST domain is underlined. N-terminal leucines (L64, L65) required for TARK1-binding are labeled. (**B**) XopN(S688A) mutant does not interact with TFT1 in yeast. Serine 688 in XopN was mutated to alanine. Yeast strain AH109 carrying pXDGATcy86(GAL4-DNA binding domain) containing XopN, and XopN(S688A) was transformed with the following PREY constructs: pGADT7(GAL4 activation domain) alone (Vector) or pGADT7 containing TFT1. Strains were spotted on nonselective (SD-LT) and selective (SD-LTH) medium and then incubated at 30°C for 3d. (**C**) XopN(S688A) and two phosphomimetic mutants, XopN(S688D) and XopN(S688E), interact with TFT1 in *N. benthamiana*. Leaves were hand-infiltrated with a suspension (8×10^8^ CFU/mL total) of two *A. tumefaciens* strains expressing TFT1-HA and XopN-6His or XopN(S688A)-6His or XopN(688D)-6His or XopN(688E)-6His. After 48 h, protein was extracted, purified by Ni^+^ affinity chromatography, and analyzed by protein gel blot analysis using anti-His and anti-HA sera. Expected protein MW: XopN-6His, S688A-6xHis, S688D-6His, and S688E-6His = 78.7 kDa; TFT1-HA = 29.3 kDa. +, protein expressed; −, vector control. (**D**) Growth of Xcv Δ*xopN* (vector), Xcv *ΔxopN* (XopN-HA), Xcv *ΔxopN* (XopN(S688A)-HA), Xcv *ΔxopN* (XopN(S688D)-HA, or Xcv *ΔxopN* (XopN(S688E)-HA in susceptible tomato VF36 leaves. Leaves were inoculated with a 1×10^5^ CFU/mL suspension of bacteria. Number of bacteria in each leaf was quantified at 0 and 10 DPI. Data points represent mean CFU/cm^2^ ± SD of four plants. Different letters at day 10 indicate statistically significant (one-way analysis of variance and Tukey's HSD test, P<0.05) differences between the samples. Vector = pVSP61. (**E**) Phenotype of tomato leaves inoculated with the strains described in (**D**). Leaves were photographed at 12 DPI. Analysis was repeated two times.

**Figure 7:**
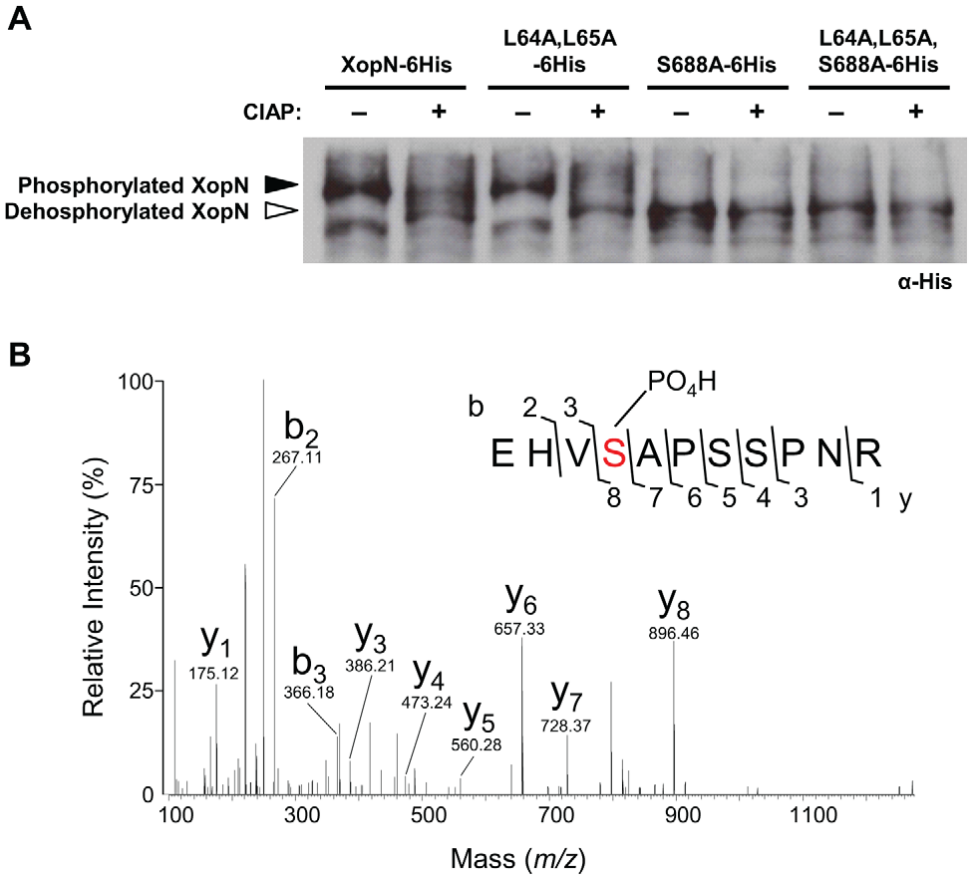
XopN is phosphorylated in plant extracts. (**A**) Phos-tag gel analysis of XopN-6His, XopN(L64A,L65A)-6His, XopN(S688A)-6His, or XopN(L64A,L65A,S688A)-6His purified from *N. benthamiana* leaves at 48 HPI by Ni^+^ affinity chromatography. Protein was treated without or with CIAP for 60 min and then separated on 8% SDS-PAGE gels containing 50 µM Mn^2+^-Phos-tag. Gels were analyzed by immunoblot analysis using anti-His sera. (**B**) MS analysis of a XopN phosphopeptide isolated from *N. benthamiana* leaf extracts. The graph shows the fragmentation spectrum of the phosphopeptide EHVSAPpSSPNR. Serine 688 is phosphorylated. Major identified b- and y- ions are labeled. The m/z value for each b- and y- ion is shown.

Examination of the C-terminal sequence of XopN revealed a putative Mode II recognition motif for 14-3-3 binding proteins [50] between amino acid residues 684–690, REHVSAP (Figure 6A). Mode II binding sites have the consensus sequence RXXXpS/TXP, where pS/pT represents phospho-serine or phospho-threonine and X can be any amino acids [50]. This suggested that serine residue 688 might be phosphorylated. To begin to address this, we first determined if XopN is phosphorylated in plant extracts using Phos-tag SDS-PAGE gels. His-tagged proteins were transiently expressed in *N. benthamiana*, purified using Ni^+^ affinity chromatography and then incubated with and without calf intestinal alkaline phosphatase (CIAP). Proteins were then separated in an 8% SDS-PAGE gel containing 50 µM Mn^2+^-Phos-tag and then analyzed by immunoblot analysis. XopN-6His treated with CIAP migrated faster in the Phos-tag gel compared to untreated XopN-6His indicating that XopN is phosphorylated in *N. benthamiana* extracts (Figure 7A).

Next we examined the phosphorylation state of XopN when S688 was mutated to alanine, XopN(S688A)-6His. We also monitored the phosphorylation state of XopN(L64A,L65A)-6His, a mutant with reduced virulence activity [28]. Leucine 64 and 65 are required to bind to TARK1 in plant extracts and to suppress PTI [28], highlighting the importance of the N-terminal domain in XopN virulence. The migration patterns of XopN(L64A,L65A)-6His with and without CIAP treatment were similar to that of XopN-6His (Figure 7A). By contrast, untreated XopN(S688A)-6His migrated similarly to the dephosphorylated form of XopN-6His, XopN(L64A,L65A)-6His, and XopN(S688A) (Figure 7A). The XopN(S688A)-6His mutant protein was generally less abundant than XopN-6His and XopN(L64A,L65A)-6His. We also examined the migration of a triple mutant, XopN(L64A,L65A,S688A)-6His. The triple mutant migrated similarly to XopN(S688A)-6His ± CIAP (Figure 7A). Phos-tag gel analysis indicates that mutation of S688 affects the extent to which the XopN polypeptide is phosphorylated in *N. benthamiana* leaf extracts.

Phosphopeptide enrichment followed by mass spectrometry (MS) was then performed with XopN-6His protein purified from *N. benthamiana* extracts to determine if S688 is phosphorylated. The phosphopeptide EHVpSAPSSPNR was identified (Figure 7B), confirming that XopN is phosphorylated in the putative 14-3-3 Mode II recognition motif at S688.

### Mutation of Serine 688 in XopN disrupts TFT1 binding in yeast but not plant extracts

To determine if phosphorylation of S688 in XopN is required for TFT1 binding, we performed a directed yeast two-hybrid analysis using DBD-XopN(S688A) as the BAIT and AD-TFT1 as the PREY. The XopN(S688A) mutant did not interact with TFT1 (Figure 6B) despite the fact that DBD-XopN(S688A) was expressed at the same level as DBD-XopN in yeast (Figure S6B). Second, we performed a *N. benthamiana* pull-down assay to determine if TFT1 can still bind the XopN(S688A) mutant in plant extracts. *N. benthamiana* leaves were infected with a suspension of two *A. tumefaciens* strains (final concentration 8×10^8^ CFU/mL) expressing TFT1-HA and vector, or XopN-6His or XopN(S688A)-6His. Pull-down analysis was then performed as described above. Unexpectedly, TFT1-HA was still detected in the pull-down with XopN(S688A)-6His, although TFT1 enrichment was less than that obtained with wild type XopN-6His (Figure 6C).

We also tested the affect of phosphomimetic mutations at S688 on XopN's binding interaction with TFT1. S688 was mutated to aspartic acid (D) or glutamic acid (E) creating the XopN(S688D)-His and XopN(S688E)-His mutants, respectively. The phosphomimetic mutants interacted with TFT1; however, as observed for the S688A mutant, less TFT1 was enriched in the pull-down using *N. benthamiana* extracts (Figure 6C). Collectively, these data indicate that mutation of S688 is not alone sufficient to disrupt or stabilize the XopN/TFT1 interaction detected in this binding assay.

### S688 is not required for XopN virulence

The discrepancy between the interaction data observed in the yeast and in plant extracts prompted us to determine the effect of the S688A, S688D, and S688E mutations on XopN-dependent virulence in susceptible VF36 tomato leaves. These mutants were independently introduced into Xcv *ΔxopN* and then the phenotypes of the resulting strains were analyzed by growth curve analysis. The mutant proteins were stably expressed in Xcv to similar levels as wild type XopN-HA (Figure S5B). Growth of Xcv *ΔxopN* expressing XopN(S688A)-HA, XopN(S688D)-HA or XopN(S688E)-HA in susceptible VF36 tomato leaves was not significantly different from that of Xcv *ΔxopN* expressing wild type XopN-HA (Figure 6D). Furthermore, the onset and severity of symptom development was similar for Xcv *ΔxopN* (XopN(S688A)-HA), Xcv *ΔxopN* (XopN(S688D)-HA), or Xcv *ΔxopN* (XopN(S688E)-HA) compared to Xcv *ΔxopN* (XopN-HA) (Figure 6E). Taken together, these data show that mutation of S688 does not impair XopN stability in Xcv or its virulence function *in planta*.

### XopN(ΔM1/M2) and PEST mutants bind TFT1

One explanation for the XopN(S688A)/TFT1 interaction *in planta* could be to the presence of additional 14-3-3 binding sites in the C-terminal region of XopN. Closer examination of this region revealed a putative Mode I 14-3-3 binding site (Figure 6A) between residues 665–670 (amino acids SSSQP) that partially conforms to the motif RXXpS/TXP [51]. We generated a XopN mutant lacking both the putative Mode I and Mode II motifs (*i.e.* XopN(ΔM1/M2)) and tested for its ability to bind TFT1 *in planta*. Additionally, we noted a putative PEST motif in XopN at amino acid residues 659–677 that overlaps with the Mode I motif. PEST motifs are generally hyperphosphorylated regions rich in proline, glutamic acid, serine, and threonine residues that are found in proteins with short half-lives [52]. We deleted the PEST sequence to determine if it affected XopN stability and/or interaction with TFT1. Both XopN(ΔM1/M2) and XopN(ΔPEST) interacted with TFT1 in the *N. benthamiana* pull-down assay (Figure S7). Moreover, deletion of the PEST motif did not significantly alter the abundance of the XopN(ΔPEST) protein nor impair its ability to rescue the virulence defect of the Xcv *ΔxopN* mutant in tomato leaves (data not shown).

### The XopN(L64A,L65A,S688A) triple mutant is impaired for TFT1 binding

Our mutation analysis suggests that the putative 14-3-3 motifs in XopN's C-terminal domain and/or phosphorylation of S688 are not the only structural determinants required for TFT1 binding specificity inside plant cells. Alternatively, the N-terminus of XopN may stabilize the XopN(S688A)/TFT1 complex in plant extracts. The N-terminus of XopN, specifically leucine 64 and 65, is required to bind TARK1 in plant extracts and to suppress PTI [28], highlighting the importance of the N-terminal domain in XopN virulence. Because we found that TARK1 can still bind XopN(S688A) in yeast (Figure S6A) and in plant extracts (Figure S8), we speculated that a TARK1 ortholog in *N. benthamiana* or another plant factor that binds to the N-terminus of XopN might strengthen the XopN(S688A)/TFT1 interaction in a protein complex.

**Figure 8:**
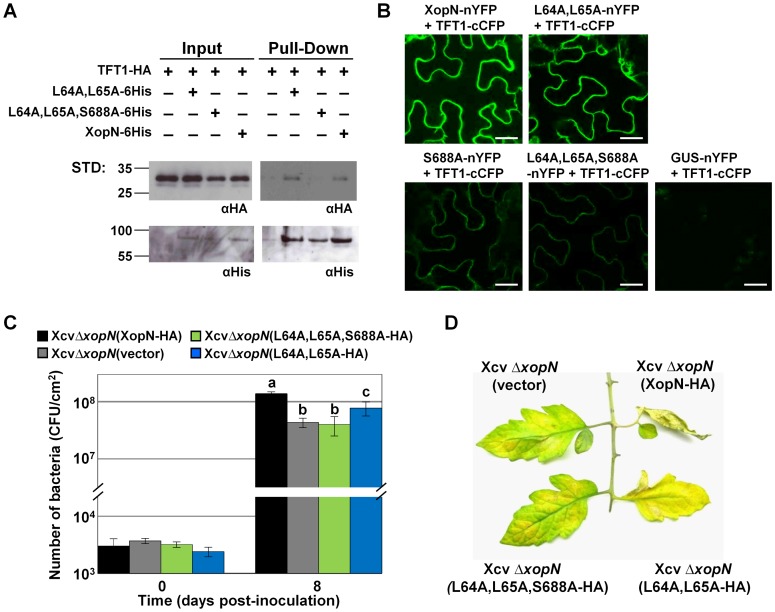
XopN L64,L65 motif and S688 are required for TFT1 binding and XopN-dependent virulence. (**A**) Pull-down analysis of TFT1-HA and XopN(L64A,L65A,S688A)-6His, XopN(L64A,L65A)-6His, or XopN-6His in *N. benthamiana*. Leaves were infiltrated with a mixed suspension of *A. tumefaciens* expressing TFT1-HA (4×10^8^ CFU/mL) and *A. tumefaciens* expressing XopN(L64A,L65A)-6His, XopN(L64A,L65A,S688A)-6His or XopN-6His (4×10^8^ CFU/mL). After 48 h, protein was extracted, purified by Ni^+^ affinity chromatography, and then analyzed by protein gel blot analysis using anti-His and anti-HA sera. Expected protein MW: TFT1-HA = 29.3 kDa; XopN(L64A,L65A)-6His, XopN(L64A,L65A,S688A)-6His and XopN-6His = 78.7 kDa. +, protein expressed; −, vector control. STD, molecular weight standard. (**B**) BiFC analysis of XopN/TFT1 interactions in *N. benthamiana*. Leaves were hand-infiltrated with a suspension (8×10^8^ CFU/mL total) of two *A. tumefaciens* strains expressing different fusion proteins (XopN-nYFP+TFT1-cCFP; L64A,L65A-nYFP+TFT1-cCFP; S688A-nYFP+TFT1-cCFP; L64A,L65A,S688A-nYFP+TFT1-cCFP; or GUS-nYFP+TFT1-cCFP) and then visualized by confocal microscopy at 48 HPI at 63X. White bar = 25 µm. (**C**) Growth of Xcv Δ*xopN* (vector), Xcv Δ*xopN* (L64A,L65A,S688A-HA), Xcv Δ*xopN* (L64A,L65A-HA), or Xcv Δ*xopN* (XopN-HA) strains in susceptible VF36 tomato leaves. Leaves were hand-infiltrated with a 1×10^5^ CFU/mL suspension of bacteria. Number of bacteria in each leaf was quantified at 0 and 8 DPI. Data points represent mean CFU/cm^2^ ± SD of three plants. Different letters at day 8 indicate statistically significant (one-way analysis of variance and Tukey's HSD test, P<0.05) differences between the samples. Vector = pVSP61. Analysis was repeated at least three times. (**D**) Phenotype of tomato leaves inoculated with the strains described in (**C**). Leaves were photographed at 12 DPI. Similar phenotypes were observed in 3 independent experiments.

To begin to test this hypothesis, we determined the role of XopN residues L64 and L65 on the XopN/TFT1 interaction. A triple point mutant XopN(L64A,L65A,S688A) was generated and its ability to bind TFT1 was assessed using the *N. benthamiana* pull-down assay. *N. benthamiana* leaves were hand-infiltrated with 6×10^8^ CFU/mL suspension of *Agrobacteria* expressing TFT1-HA alone or coexpressing TFT1-HA and XopN(L64A,L65A)-6His, XopN(L64A,L65A,S688A)-6His, or XopN-6His. Using Ni-NTA agarose beads, the respective His-tagged XopN proteins were purified by Ni^+^ affinity chromatography and analyzed by protein gel blot analysis. TFT1-HA was detected in the pull-downs with XopN-6His and XopN(L64A,L65A)-6His but not XopN(L64A,L65A,S688A)-6His (Figure 8A). These data indicate that L64, L65 and S688 are required to detect XopN/TFT1 binding in plant extracts.

To monitor the extent to which the XopN(L64A,L65A,S688A) mutant interacts with TFT1 in live plants cells, we utilized the transient bifluorescence complementation (BiFC) assay in *N. benthamiana* (Figure 8B). The non-fluorescent N-terminal domain of YFP was fused to the C-terminal domain of wild type and three mutant versions of XopN creating XopN-nYFP fusions. The non-fluorescent C-terminal domain of CFP was fused to the C-terminal domain of TFT1 creating TFT1-cCFP. For the BiFC assay, two *Agrobacteria* strains, each containing a different protein fusion construct, were mixed equally (final concentration 8×10^8^ CFU/mL) and then the mixture was hand-infiltrated into *N. benthamiana* leaves. At 48 HPI, leaf epidermal cells were imaged by confocal microscopy. As expected, co-expression of XopN-nYFP and TFT1-cCFP resulted in the emission of bright fluorescence in the cytoplasm [28] (Figure 8B). Co-expression of XopN(L64A,L65A)-nYFP and TFT1-cCFP resulted in slightly less fluorescence relative to that observed for XopN-nYFP+TFT1-cCFP (Figure 8B). By contrast, co-expression of XopN(S688A)-nYFP+TFT1-cCFP or XopN(L64A,L65A,S688A)-nYFP+TFT1-cCFP resulted in very, weak fluorescence (Figure 8B). This weak fluorescence was greater than the negative control (*i.e.* GUS-nYFP+TFT1-cCFP) or detectable background fluorescence. All proteins were expressed in *N. benthamiana* (Figure S9A). The BiFC data thus indicates that the triple mutant and the S688A mutant are still able to bind to TFT1 at some level *in planta*, however the interactions appear to be very weak. Reduced interaction between XopN(S688A)/TFT1 was detected in the pull-down assay (Figure 6C).

We also monitored interactions between TARK1 and the XopN mutants in the BiFC assay (Figure S9B,C). As expected, co-expression of TARK1-cCFP and XopN-nYFP resulted in bright fluorescence at the PM (Figure S9B). Co-expression of TARK1-cCFP and XopN(S688A)-nYFP resulted in reduced fluorescence whereas co-expression of XopN(L64A,L65A)-nYFP or XopN(L64A,L65A,S688A)-nYFP with TARK1-cCFP only resulted in background fluorescence similar to the negative control (*i.e.* GUS-nYFP+TARK1-cCFP) (Figure S9B). These data are consistent with the pull-down assays showing the mutation of L64,L65 in XopN disrupts TARK1/XopN binding whereas the S688A mutation does not (Figure S8). The BiFC data suggest that the interaction between XopN(S688A)/TARK1 may be weaker than that of XopN/TARK1; however, this was not observed in the pull-down assay.

### The XopN(L64A,L65A,S688A) triple mutant is impaired for virulence

We next assessed the impact of the triple mutant (L64A,L65A,S688A) on XopN-dependent virulence by monitoring Xcv growth and symptom development in tomato leaves as described above. Growth of Xcv *ΔxopN* expressing XopN(L64A,L65A,S688A)-HA was similar to that of the Xcv *ΔxopN* null mutant, and significantly less than that of Xcv *ΔxopN* expressing XopN-HA (Figure 8C). Growth of Xcv *ΔxopN* expressing the double mutant XopN(L64A,L65A)-HA was greater than the triple mutant but less than Xcv *ΔxopN* expressing wild type XopN-HA (Figure 8C). Leaf symptom development at 12 DPI correlated with bacterial titer. Leaves infected with Xcv *ΔxopN* (XopN-HA) exhibited severe chlorosis and tissue necrosis whereas leaves infected with Xcv *ΔxopN* (XopN(L64A,L65A,S688A)-HA) or Xcv *ΔxopN* (vector) were only slightly chlorotic (Figure 8D). Leaves infected with Xcv *ΔxopN* (XopN(L64A,L65A)-HA) exhibited an intermediate phenotype – mild chlorosis but no tissue collapse (Figure 8D). All proteins were equally expressed in Xcv *ΔxopN* (Figure S5C). These data demonstrate that residues L64, L65 and S688 in XopN are important for XopN-dependent pathogen growth and symptom development in tomato.

### XopN promotes TARK1 and TFT1 interaction *in planta*

Analysis of the XopN triple mutant (Figure 8) suggests that the N-terminus of XopN plays an important role in XopN/TFT1 binding *in planta*. It is possible that the N-terminal domain simply affects the conformation of the C-terminal domain in the context of the whole XopN polypeptide. Alternatively, but not mutually exclusive, it is possible that the N-terminus of XopN may bind a host factor, resulting in the formation of a protein complex that coordinates and/or stabilizes TFT1 binding at the C-terminus of XopN. We postulated that TARK1 might be one such host factor considering that it binds to the N-terminus of XopN [28] and binding to full-length XopN requires residues L64 and L65 (Figure S8). If TARK1 is required for XopN to bind TFT1, we reasoned that TARK1 may bind TFT1 and/or a complex comprising TARK1/XopN/TFT1 may exist *in planta*.

To begin to address this, we used the transient BiFC assay in *N. benthamiana* to directly monitor TARK1 and TFT1 protein-protein interactions inside plant cells in the presence and absence of wild type or mutant versions of XopN. For the BiFC assay, three *Agrobacteria* strains, each containing a different protein fusion construct, were mixed equally (final concentration 6×10^8^ CFU/mL) and then the mixture was hand-infiltrated into *N. benthamiana* leaves. At 48 HPI, leaf epidermal cells were imaged by confocal microscopy. The three protein fusions tested were: (1) = TFT1-Venus^N^, the non-fluorescent N-terminal domain of Venus fused to the C-terminal domain of TFT1; (2) TARK1-SCFP3A^C^, the non-fluorescent C-terminal domain of SCFP3A fused to the C-terminal domain of TARK1; and (3) wild type or mutant XopN-6His or GUS-6His, a negative control. All of the fusion proteins analyzed were expressed in *N. benthamiana* (Figure S10).

**Figure 9:**
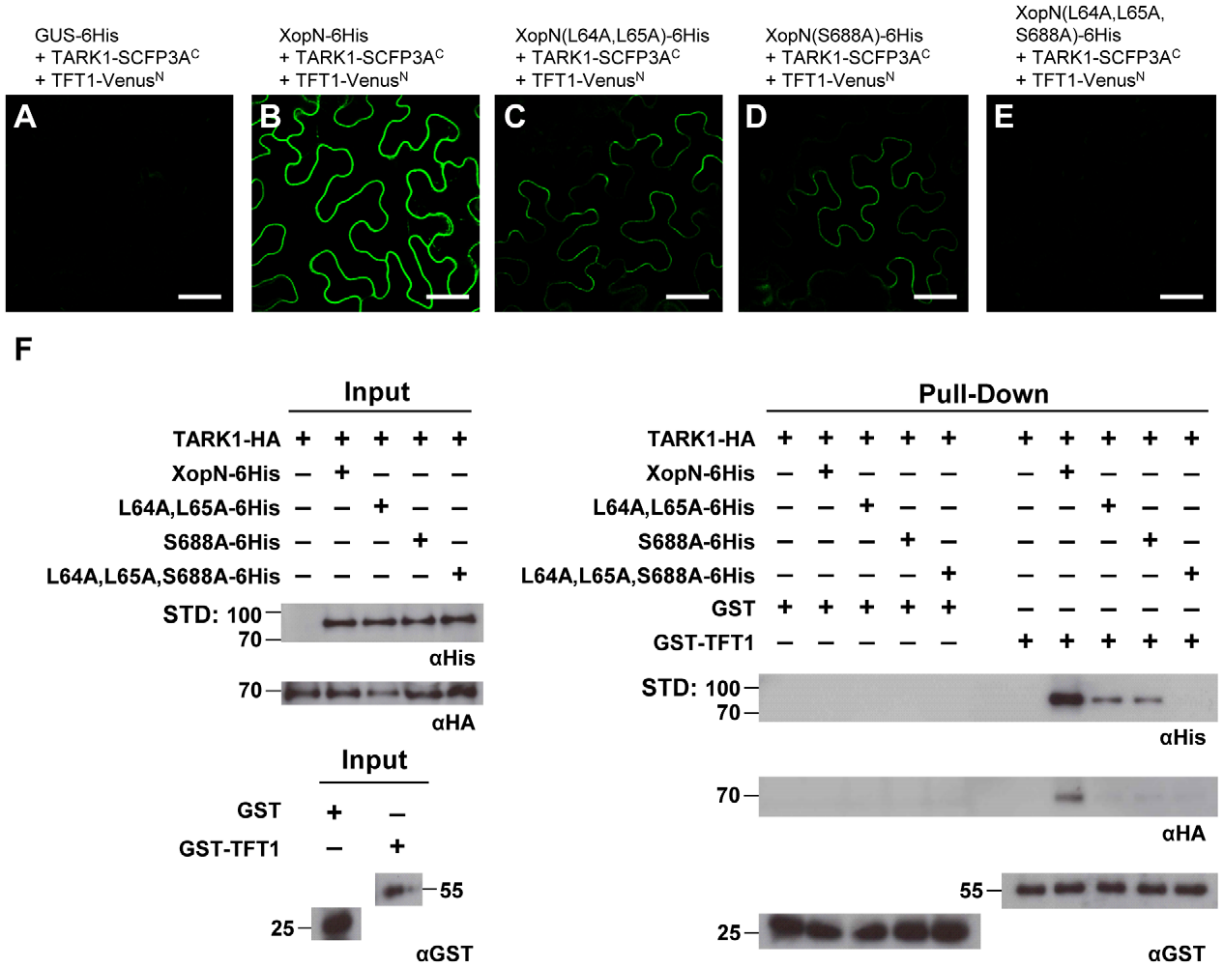
XopN promotes TARK1 and TFT1 binding in *N. benthamiana*. (**A-E**) BiFC analysis of TARK1/TFT1 interactions in *N. benthamiana* leaves in the presence of wild type and mutant XopN protein. Leaves were hand-infiltrated with a suspension (6×10^8^ CFU/mL total) of three *A. tumefaciens* strains expressing different fusion proteins: (**A**) GUS-6His+TARK1-SCFP3A^c^+TFT1-Venus^N^; (**B**) XopN-6His+TARK1-SCFP3A^c^+TFT1-Venus^N^; (**C**) XopN(L64A,L65A)-6His+TARK1-SCFP3A^c^+TFT1-Venus^N^; (**D**) XopN(S688A)-6His+TARK1-SCFP3A^c^+TFT1-Venus^N^; (**E**) XopN-(L64A,L65A,S688A)-6His+TARK1-SCFP3A^c^+TFT1-Venus^N^. Cells were visualized by confocal microscopy at 48 HPI at 63X. White bar = 40 µm. (**F**) GST-TFT1 affinity purification of TARK1 and XopN *in vitro*. GST or GST-TFT1 was incubated with *N. benthamiana* leaf extracts containing TARK1-HA ± vector, XopN-6His or mutant XopN-6His (*i.e.* XopN(L64A,L65A)-6His, XopN(S688A)-6His, or XopN(L64A,L65A,S688A)-6His). Proteins were purified using glutathione sepharose and analyzed by immunoblot analysis using anti-His, anti-HA, and anti-GST sera. Protein input levels are shown on the left. GST and GST-TFT1 pull-downs are shown on right. Expected protein MW: GST = 28 kDa; GST-TFT1 = 56.5 kDa; TARK1-HA = 67.9 KDa; XopN(L64A,L65A)-6His, XopN(L64A,L65A,S688A)-6His and XopN-6His = 78.7 kDa. +, protein expressed; −, vector control. STD, molecular weight standard.

Co-expression of TFT1-Venus^N^ and TARK1-SCFP3A^C^ in the presence of GUS-6His did not result in fluorescence above background levels (Figure 9A), indicating that these two proteins do not interact in this assay. By contrast, co-expression of TFT1-Venus^N^ and TARK1-SCFP3A^C^ in the presence of wild type XopN-6His resulted in bright fluorescence near the plasma membrane (PM) (Figure 9B). Interestingly, the fluorescence pattern at the PM resembles that observed for TARK1/XopN interaction in the BiFC assay [28] (Figure S9B). The fluorescence intensity for TFT1-Venus^N^+TARK1-SCFP3A^C^ in the presence of XopN(L64A,L65A)-6His (Figure 9C) or XopN(S688A)-6His (Figure 9D) was much weaker than that of wild type XopN-6His (Figure 9B), whereas in the presence of the triple mutant, no detectable fluorescence above background was observed (Figure 9E). The BiFC data suggest that XopN promotes TARK1/TFT1 interactions within the cell.

To confirm these findings, we developed an *in vitro* GST pull-down assay to monitor TARK1/XopN/TFT1 interactions. Recombinant GST and GST-TFT1 were expressed in *E. coli* and then purified using glutathione sepharose. TARK1-HA was coexpressed with vector, XopN-6His, XopN(L64A,L65A)-6His, XopN(S688A)-6His, or XopN(L64A,L65A,S688A)-6His in *N. benthamiana* leaves using *Agrobacteria*. Soluble protein extracts were isolated from leaves and then incubated with purified GST or GST-TFT1 in a standard GST pull-down assay. In the absence of wild type XopN, TARK1 was not purified by GST or GST-TFT1 (Figure 9F). This indicates that TARK1-HA does not physically interact with GST or GST-TFT1 under these assay conditions. By contrast, GST-TFT1 (but not GST alone) affinity purified TARK1-HA and XopN-6His when both proteins were present in the *N. benthamiana* extracts (Figure 9F). TARK1-HA was not enriched by GST-TFT1 when the plant extracts contained TARK1-HA and mutant versions of XopN-6His (*i.e.* XopN(L64A,L65A)-6His, XopN(S688A)-6His or XopN(L64A,L65A,S688A)-6His), although trace amounts of TARK1-HA were detected. Importantly, GST-TFT1 was able to pull-down XopN(L64A,L65A)-6His or XopN(S688A)-6His but not the triple mutant (Figure 9F) consistent with the binding data observed for TFT1-HA/XopN-6His (Figure 6C and 8A). These GST-TFT1 pull-down data confirm that XopN promotes TARK1/TFT1 binding *in vitro*. Mutations in XopN that impair TARK1/XopN or XopN/TFT1 binary interactions also affected the detection of a TARK1/TFT1 complex in this assay. These findings are in agreement with the BiFC data and provide additional evidence that XopN promotes TARK1/TFT1 interactions by serving as a molecular scaffold.

## Discussion

In this study, we characterized the role of the tomato 14-3-3 TFT1 in plant defense and the relevance of a putative XopN/TFT1 complex *in planta*. Given that XopN suppresses PTI during infection, we hypothesized that TFT1 might function as: (1) a positive regulator of defense enzyme activity and/or defense signal transduction, in which case XopN may suppress TFT1 activity to promote virulence; (2) a negative regulator of defense whose activity and/or stability is positively regulated by XopN binding; or (3) a “clamp” to increase and/or facilitate XopN's virulence activity within the plant cell. Our data show that *TFT1* is a PTI-induced gene (Figure 1) that is required for the expression of some PTI marker genes (Figure 3) and to inhibit Xcv growth during infection in tomato (Figure 2). Moreover, mutations that prevent XopN from binding to TFT1 in plant extracts attenuate XopN-dependent virulence in tomato (Figure 5 and 8). These findings are consistent with hypothesis 1, indicating that TFT1 is a component of the PTI machinery that is targeted by XopN during Xcv infection.

How TFT1 or any other 14-3-3 isoforms function as positive regulators of PTI is not yet clear. More is known about the role of 14-3-3s in ETI. The tomato 14-3-3 isoform 7 (TFT7) interacts with the C-terminal domain of tomato MAPKKKα and regulates PCD mediated by multiple R proteins [41], [53]. Coexpression of TFT7 and MAPKKKα results in an increase in MAPKKKα protein abundance and kinase activity [53]. TFT7 was subsequently found to interact with tomato MKK2, a MAP kinase kinase which functions downstream of MAPKKKα, revealing that TFT7 recruits multiple signaling components to mediate PCD associated with immunity [41]. In addition, plant 14-3-3s have been shown to influence R-protein function. In *Arabidopsis*, the 14-3-3 isoform λ (designated GF14λ) binds to the C-terminal domain RPW8.2 [40], an atypical R-protein that confers broad-spectrum resistance to powdery mildew disease in *Arabidopsis* [54]. Overexpression of *GF14λ* triggers localized PCD and enhanced resistance to powdery mildew, whereas reduced *GF14λ* expression compromises both basal and RPW8.2-mediated resistance [40]. In tobacco, several 14-3-3 isoforms were shown to interact with the viral resistance protein N [55]. It is speculated that the 14-3-3 acts as a scaffold between the N protein and the tobacco mosaic virus replicase in a receptor-ligand complex [55]. These studies indicate that plant 14-3-3s are functioning as clamps and scaffolds to regulate the kinetics of ETI signaling.

14-3-3s have also been implicated in the suppression of plant defense responses. *Arabidopsis* GF14λ interacts with the ankyrin repeat protein (AKR2) and ascorbate peroxidase 3 (AKR3) suggesting that GF14λ may regulate antioxidant metabolism [56]. In rice, silencing of *GF14e* led to the accumulation of reactive oxygen species, a lesion mimic phenotype and enhanced resistance to the bacterial pathogen *Xanthomonas oryzae* and fungal pathogen *Rhizoctonia solani* [57]. The client for GF14e is not known. Thus, the extent to which 14-3-3s negatively regulate plant defense signal transduction remains to be determined.

The physical association of T3S effectors with host 14-3-3s appears to be an emerging theme in host-microbe interactions [28], [42]–[44]; although, the phenotypes associated with the formation of these complexes are quite distinct. For example, ExoS recruits a 14-3-3 to enhance ExoS-dependent ADP-ribosyltransferase activity [45] and thus *Pseudomonas aeruginosa* virulence in mice [46]. AvrRxv, another Xcv effector, interacts with the tomato 14-3-3 isoform TFT9, resulting in the activation of ETI [42]. This suggests that TFT9 might be associated with a R-protein complex required for AvrRxv recognition and immunity, linking TFT9 function to ETI. By contrast, our data suggests that XopN targets TFT1 to suppress PTI in susceptible tomato plants. XopN doesn't appear to recruit TFT1 for its virulence activity because silencing *TFT1* expression in tomato resulted in increased susceptibility to Xcv *ΔxopN* infection (Figure 2). Thus, this is the first example of a T3S effector that targets a 14-3-3 to promote bacterial pathogenesis. It is likely that the HopM1 effector from *Pseudomonas syringae* may also directly target a 14-3-3 in plant cells. HopM1 action *in planta* affects the stability of the *Arabidopsis* 14-3-3 protein GF14κ during *Pseudomonas* infection [44]. The role of GF14κ in PTI remains to be determined.

To gain insight to how XopN might be interfering with TFT1 function, we attempted to localize the binding site in XopN that interacts with TFT1. Our mutation analysis indicates that the C-terminus of XopN is necessary (*i.e.* residues 605–733) and sufficient (*i.e.* residues 345–733) for binding to TFT1 in yeast and in plant extracts (Figure 4). Unfortunately, we were not able to determine if the C-terminal domain of XopN is alone sufficient for virulence. Neither the C-terminal or N-terminal domains of XopN expressed from its native promoter were stably expressed in Xcv (Figure S5C). We were however able to show that residues 605–733 in the C-terminus are required for XopN/TFT1 interaction (Figure 4D) and XopN virulence (Figure 5).

The C-terminal domain contains two regions that partially conform to Mode I (RXXpS/TXP) and Mode II (RXXXpS/TXP) consensus sites that are recognized by 14-3-3s [50], [51]. We confirmed by MS that S688 in the high stringency Mode II site is phosphorylated (Figure 7B). Mutation of S688 to alanine abolished XopN's ability to bind to TFT1 in yeast. Regardless, this mutation was not sufficient to prevent XopN from binding TFT1 or reduce XopN virulence in tomato. This raised the possibility that phosphorylation at S688 is not required for the XopN/TFT1 interaction or that other residues in the C-terminus of XopN can serve as binding sites for TFT1 *in planta*. Interestingly, we found that XopN is phosphorylated in plant extracts whereas the S688A mutant migrates like the dephosphorylated form of XopN (Figure 7A). This suggests that S688 may be the major phosphorylation site in XopN or S688 is required for XopN to be phosphorylated at other residues. We speculate that phosphorylated residues in XopN may play a role in stabilizing XopN/TFT1 interaction considering that the S688A mutant (*i.e.* the apparent dephosphorylated form) exhibited reduced binding to TFT1. Interestingly, the C-terminal region of XopN that is sufficient to bind to TFT1 is rich in serine and threonine residues, potential phosphorylation sites. Future work will examine if this region is hyperphosphorylated in an S688-dependent manner. The structural determinants that facilitate direct interaction between XopN and TFT1 thus remain to be determined. It is likely that the 14-3-3 binding site(s) in XopN will be unique considering that many 14-3-3 clients possess phosphorylation independent, non-canonical 14-3-3 motifs [37], including ExoS [45].

Upon examining the role of S688 on XopN/TFT1 binding, we discovered that the N-terminus of XopN (specifically residues L64 and L65) plays an important role in stabilizing the XopN(S688A)/TFT1 interaction. L64 and L65 are necessary for XopN to bind TARK1 [28] (Figure S8) but are only partially required for XopN-dependent virulence in tomato [28]. This suggests that the L64A,L65A mutant can bind to other host targets which may be required and/or targeted for full XopN virulence activity. Interestingly, the L64A,L65A mutant can still bind TFT1 (Figure 8). The triple mutant (L64A,L65A,S688A) however does not bind TFT1 in pull-down assays (Figure 8A). Moreover, Xcv *ΔxopN* expressing the triple mutant (L64A,L65A,S688A) behaves like the Xcv *ΔxopN* null mutant indicating that these three mutations are sufficient to abolish XopN virulence activity in tomato (Figure 8C). These data suggest that XopN binding to both TARK1 and TFT1 is important for XopN-dependent virulence.

The exact role for the N-terminus of XopN in facilitating TFT1 binding *in planta* remains to be determined; however, these findings suggest new models regarding the functional domains of XopN. For example, we postulate three models for which the N-terminal domain of XopN may promote XopN(S688A)/TFT1 binding: (1) The N-terminus of XopN may bind a host factor (*e.g.* TARK1), resulting in the formation of a protein complex that coordinates TFT1 binding at the C-terminus of XopN. (2) The N-terminus of XopN may be required to bind a TFT1 homodimer or heterodimer. 14-3-3 proteins are known to form rigid dimers with many of their clients [37]. If XopN does interact with a 14-3-3 dimer, our data would be consistent with the existence of a low affinity 14-3-3 binding site in XopN's N-terminus and a high affinity site in XopN's C-terminus. (3) The N-terminus may be required for protein-protein interactions that result in the post-translational modification (*e.g.* phosphorylation) of XopN inside the plant cell. Such modifications may influence the strength of the interactions between XopN and TFT1. These roles are not necessarily mutually exclusive and we can't yet rule out that the N-terminal domain simply affects the conformation of the C-terminal domain in the context of the whole polypeptide.

We tested the model that a TARK1/XopN/TFT1 complex exists in plant cells considering that both TARK1 and TFT1 play positive roles in PTI and XopN engages in binary interactions with both TARK1 and TFT1. BiFC analysis shows that co-expression of XopN with TARK1 and TFT1 in *N. benthamiana* results in the formation of a TARK1/TFT1 complex (Figure 9B). Importantly, mutations in XopN that impaired the formation of TARK1/XopN or XopN/TFT1 complexes in pull-down studies also impaired the formation of TARK1/TFT1 complexes in the BiFC analysis (Figure 9C–E). The existence of a TARK1/XopN/TFT1 complex was confirmed independently using an *in vitro* GST-TFT1 affinity pull-down assay (Figure 9F). Taken together, these data suggest that XopN may act as a protein bridge or scaffold to promote and/or stabilize TARK1/TFT1 complexes.

Why then does XopN promote the formation of a TARK1/TFT1 complex? We have shown that both TARK1 [28] and TFT1 are required to inhibit Xcv multiplication in tomato. Their precise roles in immunity however remain to be determined. Given that XopN suppresses PTI within the plant cell, it is likely that TARK1 and TFT1 function in PTI signaling downstream of Xcv recognition. TARK1 encodes a putative leucine-rich repeat receptor-like kinase (LRR-RLK) with a short extracellular domain (5 LRRs) and an inactive cytoplasmic kinase domain that is localized to the plant PM [28]. Based on TARK1's protein features, we speculate that TARK1 might interact with a primary pathogen recognition receptor or a membrane-associated defense complex to regulate PTI signaling. XopN binding to TARK1's kinase domain in the host cytoplasm could interfere with TARK1 protein-protein interactions, stability and/or signal transduction.

Currently, we do not know if TARK1 and TFT1 operate in the same or different immune pathways. In the absence of XopN, TARK1 and TFT1 do not appear to physically interact (Figure 9). This suggests that TARK1 is not likely a TFT1 client in uninfected plant cells. Interestingly, in the presence of XopN, TARK1/TFT1 complexes are detected at the cytoplasmic-PM interface. Moreover, XopN/TFT1 complexes appear to be restricted to the plant cytoplasm (Figure 6B) reflecting the subcellular distribution of XopN, not TFT1 [28]. It is thus tempting to speculate that XopN binding to TFT1 and/or TARK1 in binary or tertiary complexes (*i.e.* XopN/TFT1, XopN/TARK1, and TARK1/XopN/TFT1) may lead to the sequestration of inactive immune complexes at or near the cytoplasmic-PM interface. This could impact immune signaling in several ways: 1) Formation of XopN/TFT1 complexes could interfere with the assembly and regulation of bona fide TFT1-client interactions during infection. 2) Formation of XopN/TARK1 complexes could interfere with the association, dissociation, and/or post-translational modification of immune complexes at the PM. 3) Formation of TARK1/XopN/TFT1 complexes could trap TARK1 and TFT1 in incompetent signaling complexes preventing TARK1 and TFT1 from functioning in their respective immune signaling pathway(s). Future work will investigate the formation, dynamics, and relevance of TARK1/XopN/TFT1 interactions to provide insight into how PTI signaling restricts Xcv growth in tomato and the biochemical mechanism(s) by which XopN suppresses PTI.

In summary, we provide evidence that TFT1 plays a positive role in PTI in tomato and is required for the inhibition of Xcv growth. We also provide evidence that XopN directly binds to TFT1 to promote Xcv growth, indicating that TFT1 is a direct host target. Based on our mutation data, we speculate that protein interactions that occur at XopN's N-terminus with other host factors directly affect the binding affinity of the XopN/TFT1 complex. These data support a model where XopN binds to TFT1 to interfere with TFT1-client interactions that are required to limit Xcv growth in tomato.

## Materials and Methods

### Accession numbers for genes used in study

*xopN* = AM039952; *TFT1* = SGN-U580865; *TFT3* = SGN-U580900; *TFT6* = SGN-U581259; *TARK1* = SGN-U574507; *PR-1b1* = SGN-U579545; *LRR22* = SGN-U444576; *GRAS4* = SGN-U575365; *WRKY28* = SGN-U586086; *PTI5* = SGN-U571539.

### Bacterial strains, growth, and matings

Strains used in this study were as follows: *Escherichia coli* DH5α and TOP10; *Agrobacterium tumefaciens* C58C1 pCH32; *Xanthomonas campestris* pv. *vesicatoria* (Xcv) strain 85-10; and Xcv *ΔxopN*. *E. coli* and *A. tumefaciens* were grown on Luria agar medium [58] at 37°C and 28°C, respectively. Xcv strains were grown on nutrient yeast glycerol agar (NYGA) [59] at 28°C. Xcv antibiotic selection was rifampicin (Rif) 100 µg/mL, tetracycline (Tc) 10 µg/mL, and/or kanamycin (Km) 50 µg/mL. *A. tumefaciens* antibiotic selection was Tc 5 µg/mL, Km 50 µg/mL, and/or spectinomycin (Sp) 50 µg/mL. *E. coli* antibiotic selection was carbenicillin 50 µg/mL and/or Km 50 µg/mL. Vectors were mobilized from *E. coli* into Xcv and *A. tumefaciens* by standard triparental mating.

### PCR and DNA constructions

Polymerase chain reaction (PCR) was used to engineer restriction sites for construct gene fusions. PCR-generated DNA fragments were cloned into pCR-BluntII-TOPO or pENTR/D/TOPO (Invitrogen). Primer sequences used for PCR are listed in Table SI. Conditions used for PCR and cloning details will be available on request. The sequence of all DNA constructs was verified by cycle sequencing.

### Bacterial growth curves

To monitor Xcv growth *in planta*, *Solanum lycopersicum* cultivar VF36 leaves were hand-inoculated by complete infiltration of the leaf tissue with a 1×10^5^ CFU/mL suspension of bacteria in 10 mM MgCl_2_ using a needleless syringe. Leaflets of the same age on the same branch were used for each experimental test. Plants were kept under 16 h light/day at 28°C. Four leaf discs (0.5 cm^2^) per treatment per time point were ground in 10 mM MgCl_2_ and diluted and spotted onto NYGA plates in triplicate to determine bacterial load. Three or four biological replicates (*i.e.*, three or four plants) were used, and the experiment was repeated at least three times. The average bacterial titer ± SD is reported.

### Construction of XopN point mutants

The XopN(S688A, S688D, or S688E) mutants were generated with a QuikChange site-directed mutagenesis kit (Stratagene) using pCR-Blunt-II(*xopN*) as template and primer set BS32/BS33, JG684/JG685, or JG686/JG687. The XopN(L64A,L65A,S688A) mutant was generated by restriction enzyme digest of pCR-Blunt-II(*xopN(L64A,L65A)*) and pCR-Blunt-II(*xopN(S688A)*) mutants with *Xho*I. The 659 bp fragment from pCR-BluntII(*xopN(S688A)*) and 5,721 bp fragment from pCR-BluntII(*xopN(L64A,L65A)*) were gel purified (QIAGEN), ligated, and sequenced to create pCR-BluntII(*xopN(L64A,L65A,S688A)*).

### Yeast constructs, two-hybrid analysis, and protein extraction

Wild type XopN and five XopN deletion mutants (*i.e.*, N, C, M4, M5, and M6) were generated by PCR using the primer sets BS1/BS2, BS1/BS4, BS3/BS2, BS6/BS2, BS1/BS7, and BS1/BS8, respectively. XopN(S688A), XopN(L64A,L65A), and XopN(L64A,L65A,S688A) were generated by PCR using the primer set BS1/BS2 on pCR-Blunt-II(*xopN(S688A*)), pCR-Blunt-II(*xopN(L64A,L65A)*), and pCR-Blunt-II(*xopN(L64A,L65A,S688A)*), respectively. The respective PCR products were cloned into pENTR/D-TOPO, and then recombined into the pXDGATcy86 destination vector [60] via a Gateway LR reaction to create pXDGATcy86(*xopN*), pXDGATcy86(*N*), pXDGATcy86(*C*), pXDGATcy86(*M4*), pXDGATcy86(*M5*), pXDGATcy86(*M6*), pXDGATcy86(*S688A*), pXDGATcy86(*L64A,L65A*) and pXDGATcy86(*L64A,L65A,S688A*). TFT1 was generated by PCR using the primer set GB3/GB4 and cloned into pENTR/D-TOPO and then recombined into the pGADT7 destination vector via a Gateway LR reaction to create pGADT7(*TFT1*). All pXDGATcy86 vectors containing wild type or *xopN* mutants were cotransformed with pGADT7(*TFT1*) into yeast strain AH109. Yeast transformants were grown on SD-LT media and then selected on SD-LTH media at 30°C for 3 days to assess protein interaction. To isolate yeast protein, cells in log phase were pelleted, resuspended in lysis buffer (1.85M NaOH and 7% 2-mercaptoethanol) and then proteins were precipitated in 10% trichloroacetic acid. Protein pellets were washed in 1M Tris, pH 6.8 and then resuspended in 8M urea sample buffer.

### Protein gel blot analysis

Proteins were separated by SDS-PAGE and analyzed by immunoblot analysis as described [61]. Proteins were visualized by chemiluminescence using anti-HA (Covance), anti-c-myc (Covance), anti-GFP (BD Biosciences), anti-6xHis (Qiagen), and anti-XopN antibodies [28], peroxidase-conjugated secondary antibodies (Bio-Rad) and ECL reagent (GE Biosciences).

### Construction of XopN mutants in binary vectors

Wild-type and three deletions mutants (XopN_1–349_-6His, XopN_345–733_-6His, and XopN_1–604_-6His were generated by PCR using the primer sets JG282/JG285, JG282/JG283, JG284/JG285, JG282/JG656, respectively, and pCR-Blunt-II(*xopN*) as template. The genes were TOPO cloned into pCR-Blunt-II. XopN(S688A, S688D, or S688E)-6His, XopN(L64A,L65A)-6His, XopN(L64A,L65A,S688A)-6His were generated by PCR amplification of pCR-Blunt-II(*xopN(S688A, S688D, or S688E)*), pCR-Blunt-II(*xopN(L64A,L65A)*), and pCR-Blunt-II(*xopN(L64A,L65A,S688A)*) templates, respectively, using the primer sets JG282/JG285 and TOPO cloned into pCR-Blunt-II. XopN-6His, XopN_1–349_-6His, XopN_345–733_-6His, XopN_1–604_-6His, XopN(S688A, S688D, or S688E)-6His, XopN(L64A,L65A)-6His, XopN(L64A,L65A,S688A)-6His were then subcloned into the *Hind*III and *Xba*I sites of pEZRK-LCY creating pEZRK(*xopN-6His*), pEZRK(*xopN*_1–349_-*6His*), pEZRK(*xopN*_345–733_-*6His*), pEZRK(*xopN(1-604)-6His*), pEZRK(*xopN(S688A, S688D, or S688E)-6His*), pEZRK(*xopN(L64A,L65A)-6His*) and pEZRK(*xopN(L64A,L65A,S688A)-6His*), respectively. To construct binary plasmids containing one gene, *TARK1-HA* and *TFT1-HA* were independently cloned into the *Xba*I and *Sac*I sites of the pATC940 vector (a gift from Stanton B. Gelvin) containing the superpromoter [62]. To construct binary plasmids containing two genes, *TARK1-HA* and *TFT1-HA* were each sub-cloned into the *Xba*I and *Sac*I sites of pBSII(SP-T) [28], a vector containing the (*ocs*)*3mas* super-promoter (SP) and NOS terminator (T) sequences. SP-TARK1-HA-NOS and SP-TFT1-HA fragments were then sub-cloned into the *Spe*I site of the pEZRK-derived vectors creating pEZRK(*xopN-6His+TARK1-HA*), pEZRK(*xopN-6His+TFT1-HA*), pEZRK(*xopN_1–349_-6His+TARK1-HA*), pEZRK(*xopN_1–349_-6His+TFT1-HA*), pEZRK(*xopN_345–733_-6His+TARK1-HA*), pEZRK(*xopN_345–733_-6His+TFT1-HA*), pEZRK(*xopN_1–604_-6His+TARK1-HA*), pEZRK(*xopN_1–604_-6His+TFT1-HA*), pEZRK(*xopN(L64A,L65A)-6His+TARK1-HA*), pEZRK(*xopN(L64A,L65A)-6His+TFT1-HA*), pEZRK(*xopN(L64A,L65A,S688A)-6His+TARK1-HA*), pEZRK(*xopN(L64A,L65A,S688A)-6His+TFT1-HA*),and pEZRK(*xopN(S688A)-6His+TFT1-HA*). All constructs were transformed into *A. tumefaciens* strain C58C1 pCH32 for transient protein expression in *N. benthamiana*.

### *Agrobacterium*-mediated transient protein expression in *N. benthamiana*

All binary plasmids were transformed into *Agrobacterium tumefaciens* strain C58C1 pCH32 for transient protein expression in *N. benthamiana*. Strains were grown overnight at 28°C on Luria agar medium containing the appropriate antibiotics. Bacteria were collected and incubated in media (10 mM MES, pH 5.6, 10 mM MgCl_2_ and 150 µM acetosyringone; Acros Organics) 2 h before inoculation. Leaves were hand-inoculated with a suspension of either one (6×10^8^ cells/mL) or two (8×10^8^ cells/mL) strains in induction media. Plants were incubated at room temperature under continuous low light for 2 to 4 days.

### *N. benthamiana* pull-down assays

Proteins were coexpressed in *N. benthamiana* leaves via the *Agrobacterium*-mediated transient expression assay. After 48 hours, leaves were frozen in liquid N_2_ and then pulverized in extraction buffer (50 mM NaH_2_PO_4_, pH 8.0, 150 mM NaCl, 10 mM imidazole, 2% glycerol, 1% Triton X-100, 1 mM PMSF). Samples were solubilized and then centrifuged for 20 min at 12,000 *g* at 4°C. Supernatant was filtered through miracloth. Soluble extracts were incubated with 15 µL of a 50% slurry of Ni-nitrilotriacetic acid Superflow agarose (Qiagen). Agarose was recovered by centrifugation and washed three times with extraction buffer. Proteins were eluted with 50 µL of sample buffer and then analyzed by protein gel blot analysis.

### Construction of XopN mutants for expression in Xcv *ΔxopN*

Overlapping PCR was used to create a DNA fragment containing the *xopN* promoter and the *xopN* ORF containing the S688A, S688D, or S688E mutation or *xopN* ORF containing the L64A,L65A,S688A mutations. The 5′ region of *xopN* (-690 bp to +148 bp) was PCR amplified from pVSP61(*P_xopN_:xopN-HA*) using primer set JR170/JR15. The ORFs of XopN(S688A, S688D, or S688E)-HA and XopN(L64A,L65A,S688A)-HA were PCR amplified from pCR-Blunt-II(*S688A, S688D, or S688E*) and pCR-Blunt-II(*L64A,L65A,S688A*), respectively, using primer set BS1/JR227. The 5′ fragment and mutated ORFs were used as templates in overlapping PCR and the respective product was cloned into pCR-Blunt-II, creating pCR-Blunt-II(*P_xopN_:xopN(S688A, S688D, or S688E)-HA*) and pCR-Blunt-II(*P_xopN_:xopN(L64A,L65A,S688A)-HA*). The *Eco*RI fragment was then subcloned into pVSP61 to create pVSP61(*P_xopN_:xopN(S688A, S688D, or S688E)-HA*) and pVSP61(*P_xopN_:xopN(L64A,L65A,S688A)-HA*). To create pVSP61-BHI, pVSP61 was digested with *Bam*HI, treated with Klenow fragment and ligated. The *Eco*RI fragment of pCR-Blunt-II(*P_xopN_:xopN-HA*) was subcloned into pVSP61-BHI to create pVSP61-BHI(*P_xopN_:xopN-HA*). The ORF encoding XopN_1–604_ was amplified from pCR-Blunt-II(*xopN*) using the primer set BS1/KT34 to generate pCR-Blunt-II(*xopN_1–604_-HA*). pCR-Blunt-II(*xopN_1–604_-HA*) and pVSP61-BHI(*P_xopN_:xopN-HA*) were digested with *Xho*I and *Bam*HI. The 660 bp fragment from pCR-Blunt-II(*xopN_1–604_-HA*) was ligated into the pVSP61-BHI(*P_xopN_:xopN-HA*) digested with *Xho*I and *Bam*HI to create pVSP61-BHI(*P_xopN_:xopN_1–604_-HA*). All vectors were introduced into Xcv 85-10 *ΔxopN* by triparental mating.

### Virus-induced gene silencing in tomato

A 457 bp fragment of the −34 to 423 region of *TFT1* was amplified using primer set JR207/JR208, cloned into pCR8/GW, and moved into the Gateway destination binary vector pTRV2 [63] using an LR clonase reaction (Invitrogen). Similarly, a 494 bp fragment of the phytoene desaturase (*PDS*) gene was amplified and moved into pTRV2 to serve as a silencing control [63]. Binary vectors were mobilized into *A. tumefaciens* C58C1 pCH32 by triparental mating. A modified protocol [63] was used for VIGS. VF36 tomato seedlings with fully expanded cotyledons but no true leaves (approximately 10 days old) were inoculated with a mixed inoculum containing a 1.5×10^8^ CFU/mL suspension of *Agrobacteria* containing pTRV1 (contains the RNA-dependent RNA polymerase of tobacco rattle virus) and a 1.5×10^8^ CFU/mL suspension of *Agrobacteria* containing pTRV2 (contains the viral coat protein and fragments of genes for silencing), pTRV2(*TFT1*) or pTRV2(*PDS*). Seedlings were put into a growth chamber at 20°C, 80% humidity, and 16 hr of light for three weeks until *PDS* silencing symptoms (chlorosis) were observed in the control plants. Standard bacterial growth curves were performed with ∼4–5-week old vector and *TFT1*-silenced plants. Prior to infection (0 DPI), total RNA was isolated from two leaflets on the same branch for each plant line to measure *TFT1*, *TFT3*, and *TFT6* mRNA levels by Q-PCR. The same leaflets were then inoculated with a 1×10^5^ CFU/mL suspension of wild type Xcv or the Xcv *ΔhrpF* mutant. The number of bacteria in each leaflet was quantified at 0, 6, and 9 DPI. At 6 DPI, total RNA was isolated from the infected leaflets to measure *PR-1b1*, *PTI5*, *GRAS4*, *WRKY28*, and *LRR22* mRNA levels by Q-PCR.

### RNA isolation and quantitative RT-PCR

Total RNA was isolated from leaves using Trizol reagent (Invitrogen) according to manufacturer's instructions. Five µg of RNA were used for cDNA synthesis. Quantitative real-time RT-PCR was performed using the cDNA and gene-specific primers (Table S1). Each cDNA was amplified by Q-PCR using Maxima SYBR Green qPCR Master Mix (Fermentas) and the MJ Opticon 2 (Bio-Rad). *ACTIN* expression was used to normalize the expression value in each sample and relative expression values were determined against the vector control using the comparative Ct method (2^−ΔΔCt^).

### Phos-tag gel analysis

Phosphate affinity SDS-PAGE using acrylamide-pendant Phos-tag (Wako Pure Chemicals) was performed as described by manufacturer to detect phosphorylated XopN mobility shifts in plant extracts. Wild type and mutant XopN-6His proteins were transiently expressed and purified using Ni-NTA chromatography as described above for the pull-down assays using modified extraction buffer (50 mM NaH_2_PO_4_, pH 8.0, 150 mM NaCl, 10 mM imidazole, 2% glycerol, 1% Triton X-100, 1 mM EDTA, 1X protease inhibitor cocktail, 5 mM NaF, and 2 mM Na_3_VO_4_) and wash buffer (50 mM NaH_2_PO_4_, pH 8.0, 150 mM NaCl, 10 mM imidazole, 2% glycerol, 1% Triton X-100). Purified proteins were incubated with buffer (50 mM Tris-HCl, pH 7.9, 100 mM NaCl, 10 mM MgCl_2_, 1 mM DTT) or buffer and 20 units of calf intestinal alkaline phosphatase for 60 min. Proteins were separated by 8% SDS-PAGE with 50 µM Mn^2+^-Phos-tag and then analyzed by protein gel blot analysis using anti-His sera.

### Phosphopeptide enrichment and mass spectrometry

XopN-6His was transiently expressed in *N. benthamiana* leaves using *Agrobacteria*-mediated transformation. Protein was solubilized from 10 g of leaves using buffer containing 100 mM sodium phosphate (pH 8), 10 mM Tris (pH 8), 8M urea, 1% CHAPS, 5 mM NaF, 2 mM Na_3_VO_4_, and protease inhibitor cocktail (Sigma). XopN-6His protein (∼5 µg) purified using Ni-nitrilotriacetic acid Superflow agarose (Qiagen) was excised from a Coomassie-stained SDS-PAGE gel and then digested with trypsin [64]. Phosphopeptides were enriched using TiO_2_ followed by HPLC and MS. The enrichment protocol was performed using titansphere phos-TiO tips (GL Sciences) and methodology as reported [65]. Peptides were separated on a Proxeon nano HPLC (Thermo Fisher) using a self packed fused silica column with a ID of 75 µM, packed with a 3 µM C18 material (Peeke Scientific). Solvent A consisted of 99.4% water/0.6% acetic acid and solvent B was 98.4% acetonitrile/1% water/0.6% acetic acid. Flow rate was 300 nL/min infused into the MS using a Proxeon source with a potential of 2.2 kV. The MS was a LTQ Orbitrap Velos, set in data dependent acquisition mode to perform both HCD and ETD on the top 3 most intense precursor ions. MS/MS on charge states 2^+^ and higher were acquired. The data was analyzed using Sequest on a Sorcerer platform. Xcalibur software (Thermo Fisher) was used to manually inspect the data and produce ion chromatograms. A custom database was used to mitigate search time.

### Bifluorescence complementation assay

To study XopN/TARK1 or XopN/TFT1 interactions, binary BiFC-Gateway destination vectors pXNGW, pNXGW, pCXGW, and pXCGW were used as previously described [28]. To study TARK1/TFT1 interactions, binary BiFC-Gateway destination vectors were obtained from Dr. Jörg Kudla [66]. Gateway LR reactions were performed with pENTR(*TFT1*) and pDEST-^GW^VYNE to create pDEST-(*TFT1-VenusN*) and pENTR(*TARK1*) and p(MAS)DEST-^GW^SCYCE to create p(MAS)DEST-*TARK1-SCFP3A^c^*). Each BiFC binary vector was transformed into *A. tumefaciens* C58C1 pCH32. The resulting two strains were mixed equally with a third C58C1 pCH32 strain containing pEZRK(*GUS-6His*), pEZRK(*xopN-6His*), pEZRK(*xopN(L64A,L65A*)*-HA*), pEZRK(*xopN(L64A,L65A,S688A*)*-HA*), or pEZRK(*xopN(S688A*)*-HA*) (final concentration 6×10^8^ CFU/mL) and then infiltrated into *N. benthamiana* leaves. At ∼48 HPI, leaf discs were placed on a slide and visualized using a 20× and 63× water immersion objective lens on a Leica TCS SP5 confocal microscope with Leica LAS AF software. Proteins were excited at 488 nm by an argon laser and emitted light was captured at 510 nm.

### GST-TFT1 affinity purification assay

Plasmids pDEST15(GST) or pDEST15(GST-TFT1) were transformed into E. coli BL21(DE3) pLysS for GST or GST-TFT1 protein expression. GST and GST-TFT1 were purified by Glutathione Sepharose 4B (GE healthcare) as described [14]. GST or GST-TFT1 bound beads were washed three times with plant extraction buffer (50 mM Tris (pH 8), 150 mM NaCl, 2% glycerol, 1% Triton X-100, and protease inhibitor cocktail (Sigma)). TARK1-HA and vector, XopN-6His, XopN(L64A,L65A)-6His, XopN(S688A)-6His, or XopN(L64A,L65A,S688A)-6His were coexpressed in *N. benthamiana* leaves using *Agrobacteria*-mediated transformation. After 48 hours, proteins were solubilized in plant extraction buffer and then incubated with GST or GST-TFT1 bound beads for 2 hours at 4°C. Beads were washed three times with extraction buffer. Purified proteins were analyzed by gel blot analysis using anti-HA sera, anti-GST sera, and anti-His sera.

## Supporting Information

Figure S1 Relative *TFT1*, *TFT3* and *TFT6* mRNA levels in the control (TRV2) and *TFT1* silenced (TRV2-TFT1) tomato lines used in **Figure 2**. Total RNA isolated from leaves prior to growth curve analysis was used for Q-PCR to monitor (**A**) *TFT1*, (**B**) *TFT3*, and (**C**) *TFT6* mRNA levels in TRV2 or TRV2-TFT1 tomato lines inoculated with Xcv, Xcv *ΔxopN*, or Xcv *ΔhrpF* at day 0. *Actin* mRNA expression was used to normalize the expression value in each sample. Error bars indicate SD for four plants.
(TIF)

Figure S2 Relative *TFT1* mRNA levels in the control (TRV2) and *TFT1*-silenced (TRV2-TFT1) tomato lines used in **Figure 3**. Total RNA isolated from infected leaves at 6 HPI was used for Q-PCR. *Actin* mRNA expression was used to normalize the expression value in each sample. Error bars indicate SD for four plants.
(TIF)

Figure S3 Protein gel blot analysis of proteins isolated from the yeast strains described in **Figure 4B**. Total protein was extracted from yeast cells and then examined by protein gel blot analysis using GAL4-DBD or HA antisera. Yeast strains analyzed were AH109 carrying pXDGATcy86 (vector, *xopN*, *xopN(N)*, *xopN(C)*, *xopN(M4)*, *xopN(M5)*, or *xopN(M6)*) and pGADT7(vector or *TFT1*). The expected molecular weights for GAL4-DBD fused to XopN, XopN(N), XopN(C), XopN(M4), XopN(M5), and XopN(M6) are approximately 97, 56, 60,74, 83, and 74 kDa, respectively. The expected molecular weight for GAL4-AD-HA fused to TFT1 is 52 kDa. Red arrowheads label the corresponding proteins. STD, molecular weight standard shown in kDa.
(TIF)

Figure S4 XopN(1–349)-6xHis associates with TARK1-HA. Pull-down analysis of TARK1-HA and XopN-6His, XopN(1–349)-6His, or XopN(345–733)-6His transiently over-expressed in *N. benthamiana* leaves using *Agrobacteria*. Leaves were hand-infiltrated with a 6×10^8^ CFU/mL suspension of *A. tumefaciens* co-expressing TARK1-HA, and XopN-6His, XopN(1–349)-6His, or XopN(345–733)-6His and TARK1-HA. After 48 hours, protein was extracted, purified by Ni^+^ affinity chromatography, and then analyzed by protein gel blot analysis using anti-His and anti-HA sera. Expected protein MW: TARK1-HA = 67.9 kDa; XopN-6xHis = 78.7 kDa; XopN(1–349)-6His = 38.0 kDa; XopN(345–733)-6His = 42.0 kDa. +, protein expressed; −, vector control. STD, molecular weight standard shown in kDa.
(TIF)

Figure S5 Protein gel blot analysis of wild-type XopN-HA or XopN mutants in Xcv Δ*xopN* cell extracts. (**A**) Protein expression levels of XopN-HA or XopN(1–604)-HA in Xcv *ΔxopN* cell extracts for data shown in **Figure 5**. (**B**) Protein expression levels of XopN-HA, XopN(S688A)-HA, XopN(S688D)-HA, or XopN(S688E)-HA in Xcv *ΔxopN* cell extracts for data shown in **Figure 6D,E**. (**C**) Protein expression levels of XopN-HA or a series of XopN mutant proteins in Xcv *ΔxopN* cell extracts for data shown in **Figure 8C,D**. Xcv strains were grown overnight at 28°C on nutrient yeast glycerol agar (NYGA) medium containing the appropriate antibiotics. Bacteria were collected and incubated in Minimal Media (7.5 mM (NH_4_)_2_SO_4_, 0.1 M KH_2_PO_4_ (pH 7.0), 2 mM Na-Citrate, 0.3% casein amino acid hydrolysate, 10 mM sucrose, 1 mM MgSO_4_, 5×10^−5^% thiamine) 12 h at 28°C with shaking. Cells were collected and washed once with 10 mM MgCl_2_. A 4 mL bacterial culture (4×10^8^ CFU/mL) MA media pH 5.4 was grown 4.5 h with shaking at 28°C. Cells were collected, resuspended in 100 µL urea sample buffer, and then analyzed by gel blot analysis using anti-HA sera. Expected protein MW: XopN-HA, L64A,L65A-HA, S688A-HA, S688D-HA and S688E-HA = 78.7 kDa; XopN(1–604)-HA = 65.2 kDa; N-term-HA = 38.3 kDa; C-term-HA = 48.0 kDa. Vector = pVSP61. STD, molecular weight standard shown in kDa.
(TIF)

Figure S6 TARK1 and TFT1 interaction with XopN(L64A,L65A,S688A) triple mutant in yeast. (**A**) Yeast strain AH109, pXDGATcy86(GAL4-DNA binding domain) containing XopN, XopN(L64A,L65A), XopN(S688A), or XopN(L64A,L65A,S688A) were independently transformed with the following PREY constructs: pGADT7(GAL4 activation domain) alone (Vector) or pGADT7 containing TARK1CD or TFT1. Strains were spotted on nonselective (SD-LT) and selective (SD-LTH) media and then incubated at 30°C for 3d. (**B**) Protein gel blot analysis of proteins isolated from the yeast strains described (**A**). Total protein was extracted from yeast cells and then examined by protein gel blot analysis using GAL4-DBD or HA antisera. Yeast strains analyzed were AH109 carrying pXDGATcy86 (vector, *xopN*, *xopN(L64A,L65A)*, *xopN(S688A)*, or *xopN(L64A,L65A,S688A)*) and pGADT7(vector or *TARK1CD*). The expected molecular weight for each GAL4-DBD fused to XopN and point mutants is ∼97 kDa, and GAL4-AD-HA fused to TARK1CD is ∼62 kDa. Red arrowheads label the corresponding proteins. STD, molecular weight standard shown in kDa.
(TIF)

Figure S7 The putative 14-3-3 binding sites and PEST motif are not required for TFT1 binding *in planta*. (**A**) XopN-ΔM1/M2-6His and (**B**) XopN-ΔPEST-6His interact with TFT1 in *N. benthamiana*. Leaves were hand-infiltrated with a 6×10^8^ CFU/mL suspension of *A. tumefaciens* co-expressing TFT1-HA and XopN-6His, XopN-ΔM1/M2-6His, and XopN-ΔPEST-6His. After 48 h, protein was extracted, purified by Ni^+^ affinity chromatography, and then analyzed by protein gel blot analysis using anti-His and anti-HA sera. Expected protein MW: XopN-6His = 78.7 kDa; XopN-ΔPEST-6His = 76.1 kDa; XopN-ΔM1/M2-6His = 76.6 kDa; TFT1-HA = 29.3 kDa.
(TIF)

Figure S8 XopN(S688A)-6His interacts with TARK1-HA in pull-down assay. Pull-down analysis of TARK1-HA and XopN-6His, XopN(L64A,L65A)-6His, or XopN(S688A)-6His transiently over-expressed in *N. benthamiana* leaves using *Agrobacteria*. Leaves were hand-infiltrated with a 6×10^8^ CFU/mL suspension of *A. tumefaciens* expressing TARK1-HA or co-expressing TARK1-HA and XopN-6His, XopN(L64A,L65A)-6His, or XopN(S688A)-6His. After 48 hours, protein was extracted, purified by Ni^+^ affinity chromatography, and then analyzed by protein gel blot analysis using anti-His and anti-HA sera. Expected protein MW: TARK1-HA = 67.9 kDa; XopN-6His, XopN(L64A,L65A)-6His, XopN(S688A)-6His = 78.7 kDa. +, protein expressed; −, vector control. STD, molecular weight standard shown in kDa.
(TIF)

Figure S9 Protein gel blot analysis and confocal microscopy for BiFC analyses. (**A**) Protein gel blot analysis of the BiFC assay monitoring XopN/TFT1 interactions shown in **Figure 8B**. Anti-XopN, anti-His and anti-GFP sera were used. (**B**) BiFC assay of XopN/TARK1 interactions in *N. benthamiana* leaves. Leaves were hand-infiltrated with a 8×10^8^ CFU/mL total suspension of two *A. tumefaciens* strains expressing different fusion proteins (*i.e.* XopN-nYFP+TARK1-cCFP; L64A,L65A-nYFP+TARK1-cCFP; S688A-nYFP+TARK1-cCFP; L64A,L65A,S688A+TARK1-cCFP; or negative control GUS-nYFP+TARK1-cCFP) and then visualized by confocal microscopy at 48 HPI at 63X. White bar = 25 µm. (**C**) Protein gel blot analysis of the BiFC assay in (**B**) above. Anti-XopN, anti-His and anti-GFP sera were used.
(TIF)

Figure S10 Protein gel blot analysis for TARK1/TFT1 BiFC assays shown in **Figure 9A-E**. Proteins were isolated from infected *N. benthamiana* leaves at 48 HPI and then analyzed by gel blot analysis using anti-His, anti-HA, and anti-c-Myc sera. Lane 1: XopN-6His+TARK1-SCFP3A^c^+TFT1-Venus^N^; Lane 2: XopN-(L64A,L65A)-6His+TARK1-SCFP3A^c^+TFT1-Venus^N^; Lane 3: XopN-(S688A)-6His+TARK1-SCFP3A^c^+TFT1-Venus^N^; Lane 4: XopN-(L64A,L65A,S688A)-6His+TARK1-SCFP3A^c^+TFT1-Venus^N^; and Lane 5: GUS-6His+TARK1-SCFP3A^c^+TFT1-Venus^N^. Venus^N^ domain has the c-Myc epitope. SCFP3A^c^ domain has the HA epitope.
(TIF)

Table S1 List of primers used in this study.
(DOC)
