# Supplementary figures and images for: Tomato TFT1 Is Required for PAMP-Triggered Immunity and Mutations that Prevent T3S Effector XopN from Binding to TFT1 Attenuate Xanthomonas Virulence
Source: PLoS Pathog. 2012 Jun 14;8(6):e1002768. doi: 10.1371/journal.ppat.1002768 (PMC3375313; doi:10.1371/journal.ppat.1002768)

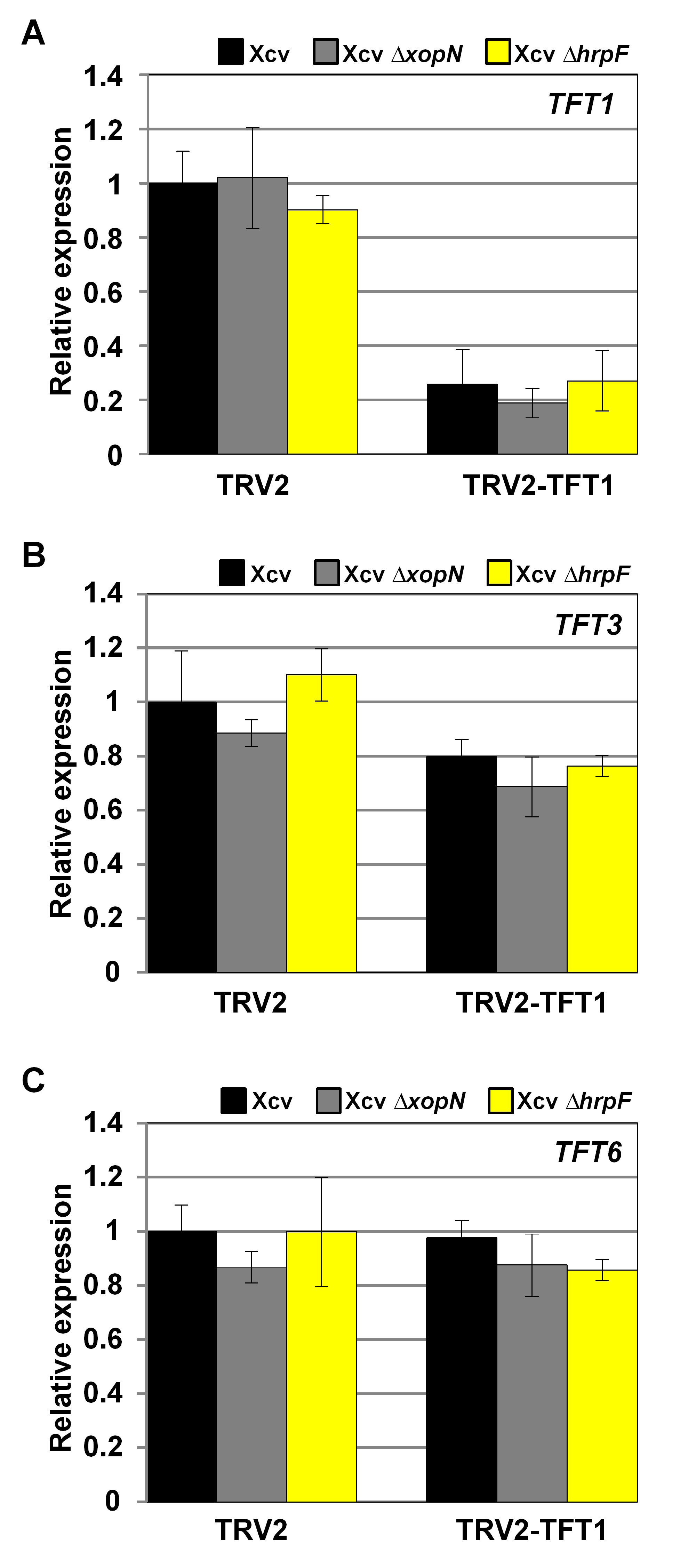

Supplement: Figure S1 — Relative TFT1, TFT3 and TFT6 mRNA levels in the control (TRV2) and TFT1 silenced (TRV2-TFT1) tomato lines used in Figure 2 . Total RNA isolated from leaves prior to growth curve analysis was used for Q-PCR to monitor (A) TFT1, (B) TFT3, and (C) TFT6 mRNA levels in TRV2 or TRV2-TFT1 tomato lines inoculated with Xcv, Xcv ΔxopN, or Xcv ΔhrpF at day 0. Actin mRNA expression was used to normalize the expression value in each sample. Error bars indicate SD for four plants. (TIF) [file ppat.1002768.s001.tif]

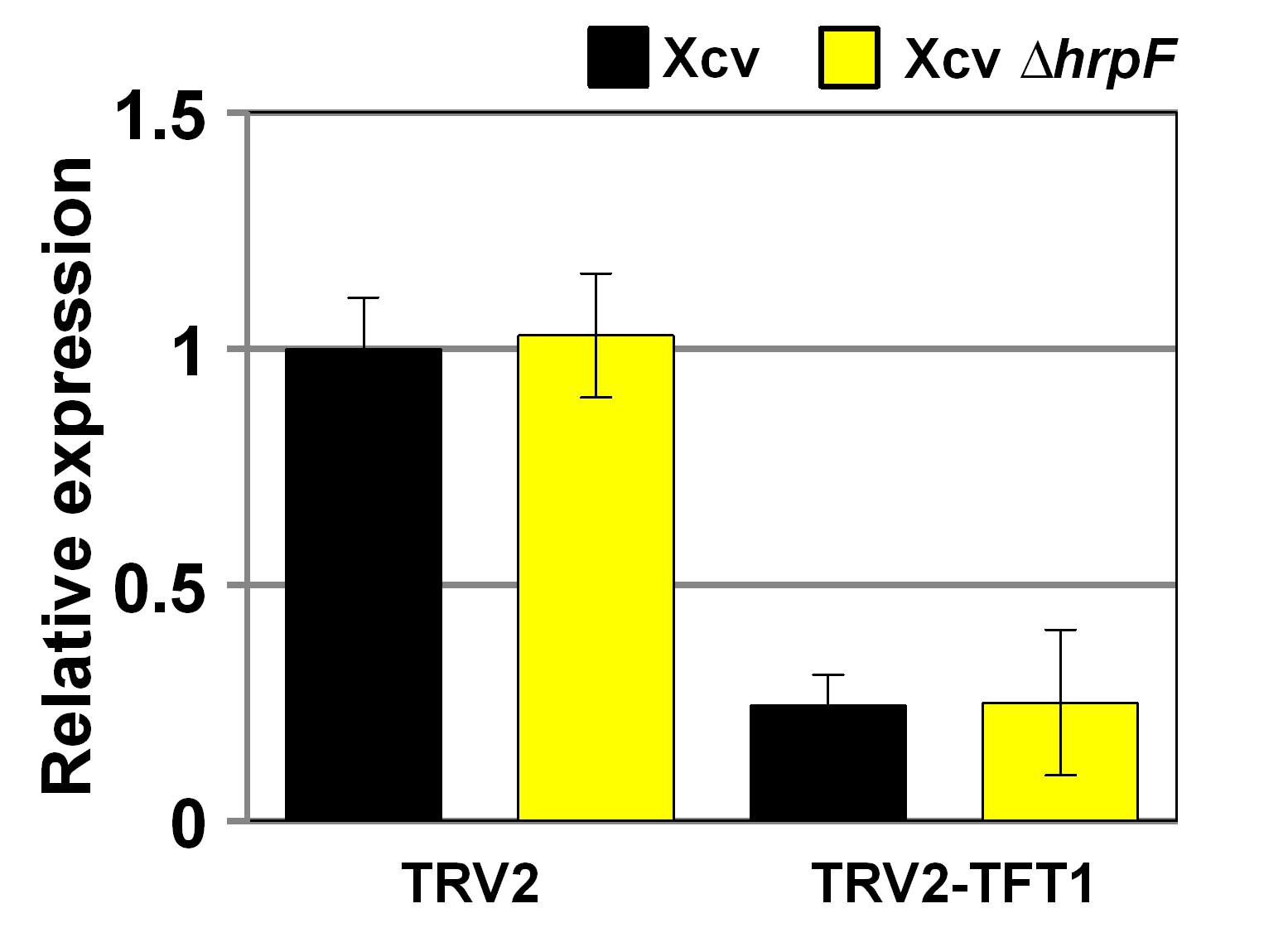

Supplement: Figure S2 — Relative TFT1 mRNA levels in the control (TRV2) and TFT1-silenced (TRV2-TFT1) tomato lines used in Figure 3 . Total RNA isolated from infected leaves at 6 HPI was used for Q-PCR. Actin mRNA expression was used to normalize the expression value in each sample. Error bars indicate SD for four plants. (TIF) [file ppat.1002768.s002.tif]

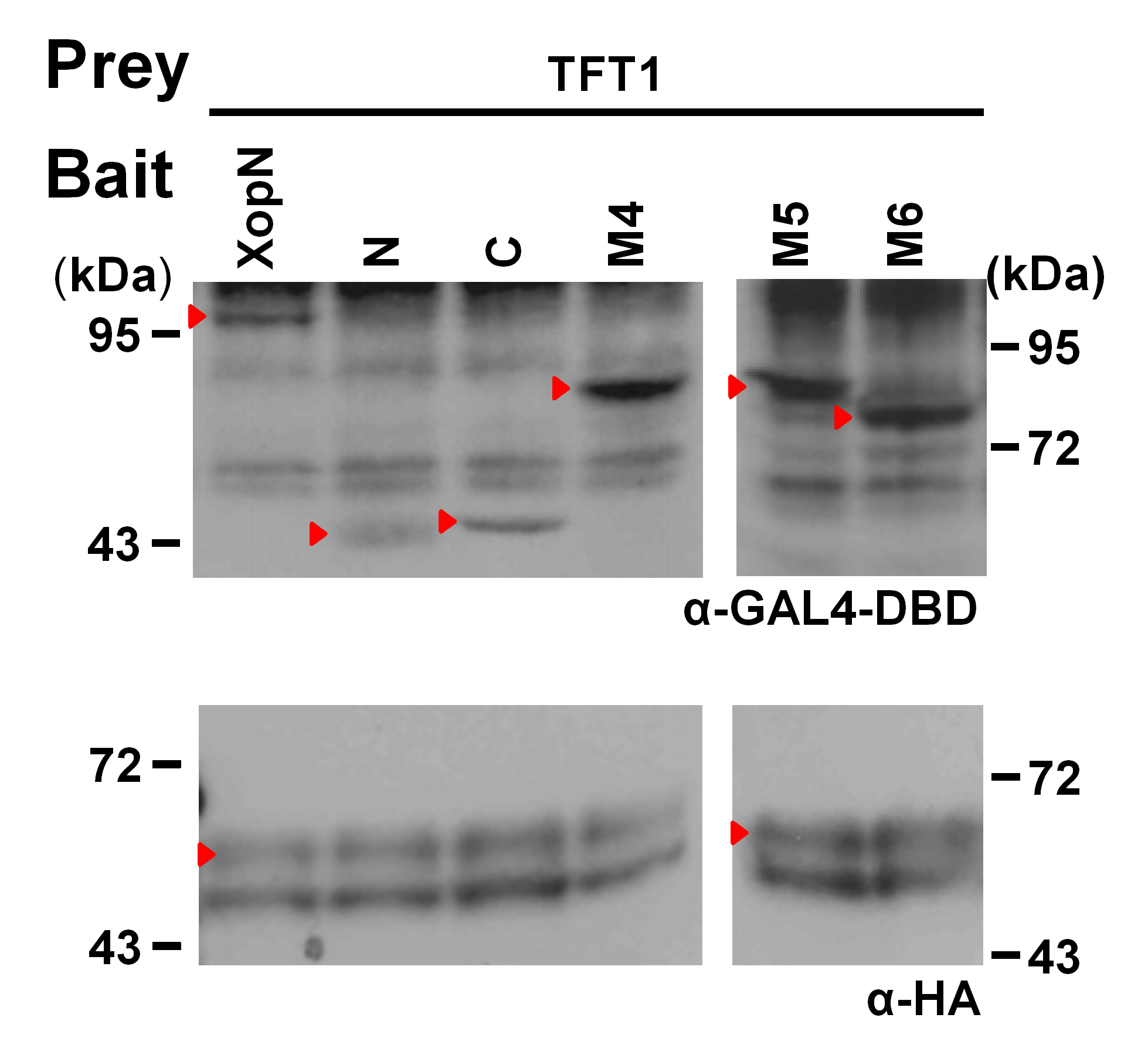

Supplement: Figure S3 — Protein gel blot analysis of proteins isolated from the yeast strains described in Figure 4B . Total protein was extracted from yeast cells and then examined by protein gel blot analysis using GAL4-DBD or HA antisera. Yeast strains analyzed were AH109 carrying pXDGATcy86 (vector, xopN, xopN(N), xopN(C), xopN(M4), xopN(M5), or xopN(M6)) and pGADT7(vector or TFT1). The expected molecular weights for GAL4-DBD fused to XopN, XopN(N), XopN(C), XopN(M4), XopN(M5), and XopN(M6) are approximately 97, 56, 60,74, 83, and 74 kDa, respectively. The expected molecular weight for GAL4-AD-HA fused to TFT1 is 52 kDa. Red arrowheads label the corresponding proteins. STD, molecular weight standard shown in kDa. (TIF) [file ppat.1002768.s003.tif]

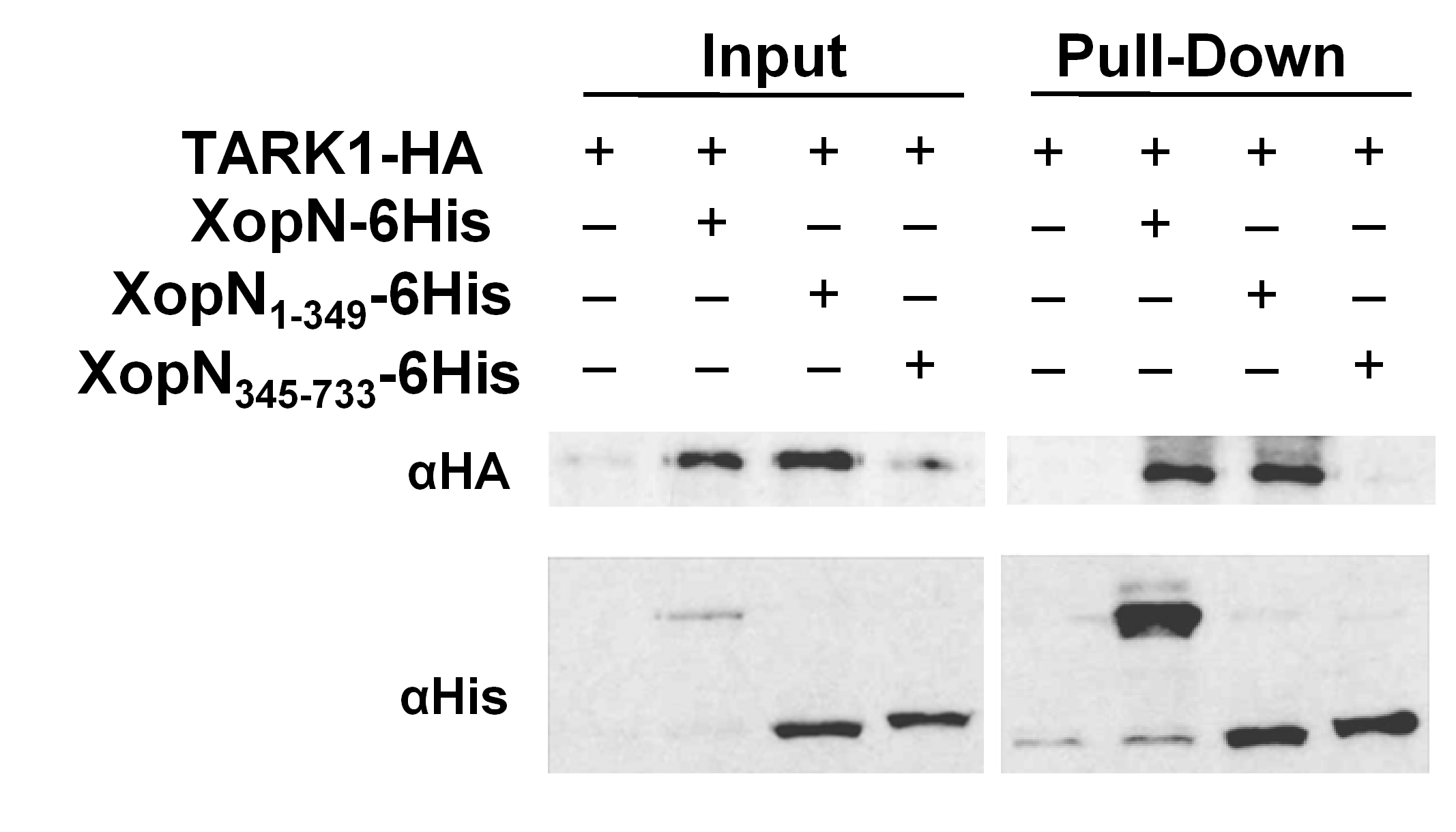

Supplement: Figure S4 — XopN(1–349)-6xHis associates with TARK1-HA. Pull-down analysis of TARK1-HA and XopN-6His, XopN(1–349)-6His, or XopN(345–733)-6His transiently over-expressed in N. benthamiana leaves using Agrobacteria. Leaves were hand-infiltrated with a 6×10^8^ CFU/mL suspension of A. tumefaciens co-expressing TARK1-HA, and XopN-6His, XopN(1–349)-6His, or XopN(345–733)-6His and TARK1-HA. After 48 hours, protein was extracted, purified by Ni+ affinity chromatography, and then analyzed by protein gel blot analysis using anti-His and anti-HA sera. Expected protein MW: TARK1-HA = 67.9 kDa; XopN-6xHis = 78.7 kDa; XopN(1–349)-6His = 38.0 kDa; XopN(345–733)-6His = 42.0 kDa. +, protein expressed; −, vector control. STD, molecular weight standard shown in kDa. (TIF) [file ppat.1002768.s004.tif]

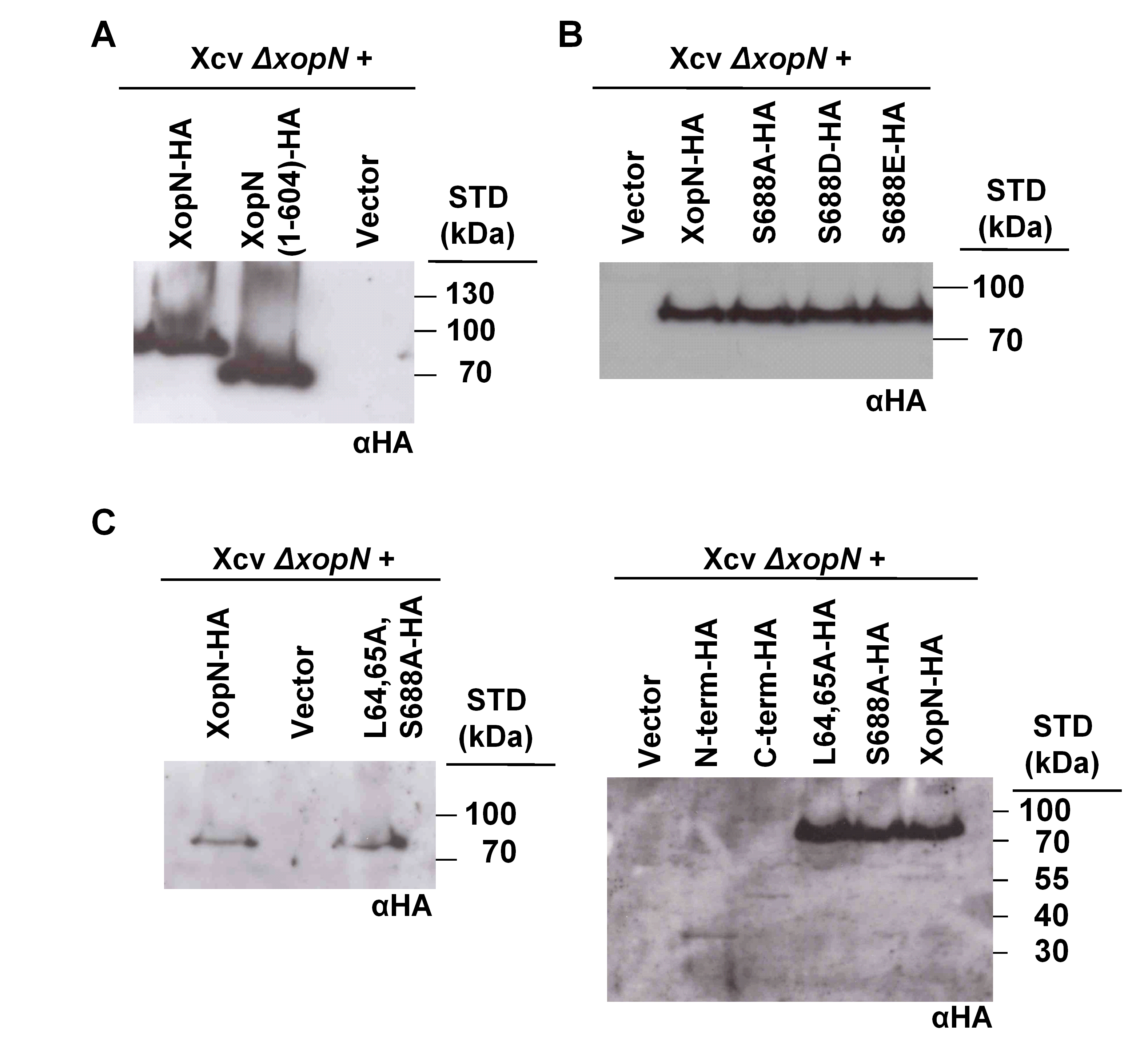

Supplement: Figure S5 — Protein gel blot analysis of wild-type XopN-HA or XopN mutants in Xcv ΔxopN cell extracts. (A) Protein expression levels of XopN-HA or XopN(1–604)-HA in Xcv ΔxopN cell extracts for data shown in Figure 5 . (B) Protein expression levels of XopN-HA, XopN(S688A)-HA, XopN(S688D)-HA, or XopN(S688E)-HA in Xcv ΔxopN cell extracts for data shown in Figure 6D,E . (C) Protein expression levels of XopN-HA or a series of XopN mutant proteins in Xcv ΔxopN cell extracts for data shown in Figure 8C,D . Xcv strains were grown overnight at 28°C on nutrient yeast glycerol agar (NYGA) medium containing the appropriate antibiotics. Bacteria were collected and incubated in Minimal Media (7.5 mM (NH4)2SO4, 0.1 M KH2PO4 (pH 7.0), 2 mM Na-Citrate, 0.3% casein amino acid hydrolysate, 10 mM sucrose, 1 mM MgSO4, 5×10−5% thiamine) 12 h at 28°C with shaking. Cells were collected and washed once with 10 mM MgCl2. A 4 mL bacterial culture (4×10^8^ CFU/mL) MA media pH 5.4 was grown 4.5 h with shaking at 28°C. Cells were collected, resuspended in 100 µL urea sample buffer, and then analyzed by gel blot analysis using anti-HA sera. Expected protein MW: XopN-HA, L64A,L65A-HA, S688A-HA, S688D-HA and S688E-HA = 78.7 kDa; XopN(1–604)-HA = 65.2 kDa; N-term-HA = 38.3 kDa; C-term-HA = 48.0 kDa. Vector = pVSP61. STD, molecular weight standard shown in kDa. (TIF) [file ppat.1002768.s005.tif]

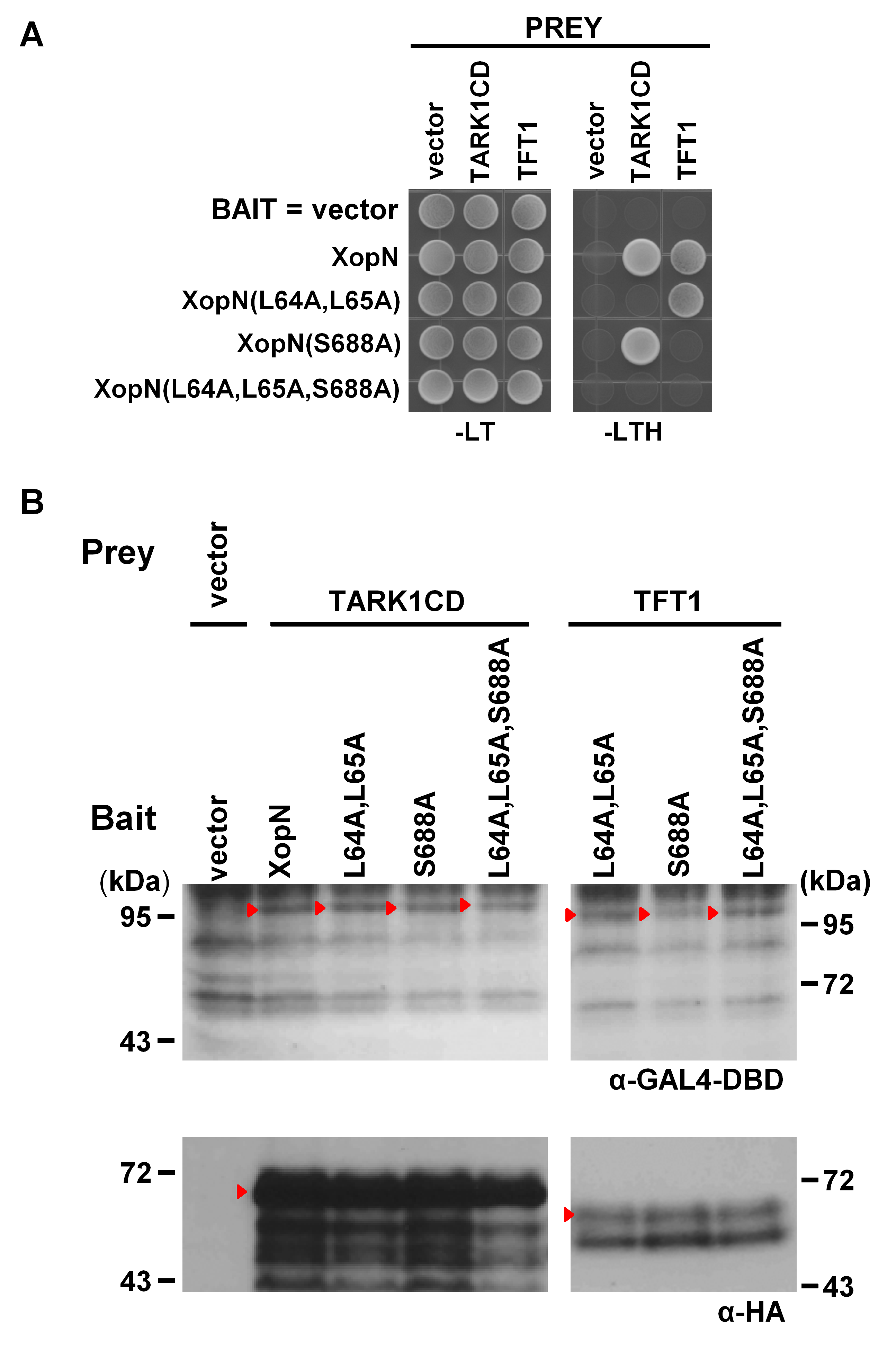

Supplement: Figure S6 — TARK1 and TFT1 interaction with XopN(L64A,L65A,S688A) triple mutant in yeast. (A) Yeast strain AH109, pXDGATcy86(GAL4-DNA binding domain) containing XopN, XopN(L64A,L65A), XopN(S688A), or XopN(L64A,L65A,S688A) were independently transformed with the following PREY constructs: pGADT7(GAL4 activation domain) alone (Vector) or pGADT7 containing TARK1CD or TFT1. Strains were spotted on nonselective (SD-LT) and selective (SD-LTH) media and then incubated at 30°C for 3d. (B) Protein gel blot analysis of proteins isolated from the yeast strains described (A). Total protein was extracted from yeast cells and then examined by protein gel blot analysis using GAL4-DBD or HA antisera. Yeast strains analyzed were AH109 carrying pXDGATcy86 (vector, xopN, xopN(L64A,L65A), xopN(S688A), or xopN(L64A,L65A,S688A)) and pGADT7(vector or TARK1CD). The expected molecular weight for each GAL4-DBD fused to XopN and point mutants is ∼97 kDa, and GAL4-AD-HA fused to TARK1CD is ∼62 kDa. Red arrowheads label the corresponding proteins. STD, molecular weight standard shown in kDa. (TIF) [file ppat.1002768.s006.tif]

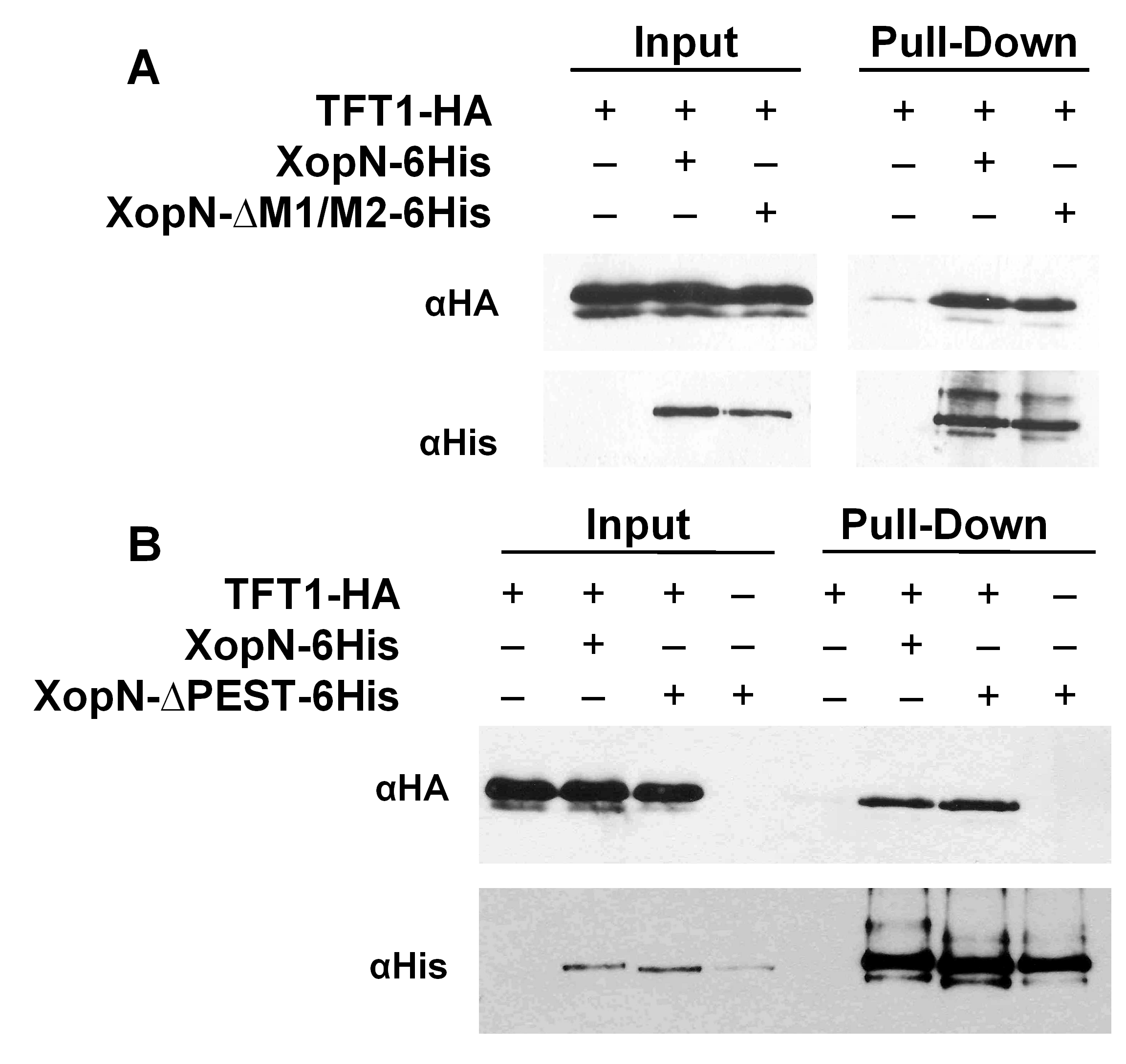

Supplement: Figure S7 — The putative 14-3-3 binding sites and PEST motif are not required for TFT1 binding in planta. (A) XopN-ΔM1/M2-6His and (B) XopN-ΔPEST-6His interact with TFT1 in N. benthamiana. Leaves were hand-infiltrated with a 6×10^8^ CFU/mL suspension of A. tumefaciens co-expressing TFT1-HA and XopN-6His, XopN-ΔM1/M2-6His, and XopN-ΔPEST-6His. After 48 h, protein was extracted, purified by Ni+ affinity chromatography, and then analyzed by protein gel blot analysis using anti-His and anti-HA sera. Expected protein MW: XopN-6His = 78.7 kDa; XopN-ΔPEST-6His = 76.1 kDa; XopN-ΔM1/M2-6His = 76.6 kDa; TFT1-HA = 29.3 kDa. (TIF) [file ppat.1002768.s007.tif]

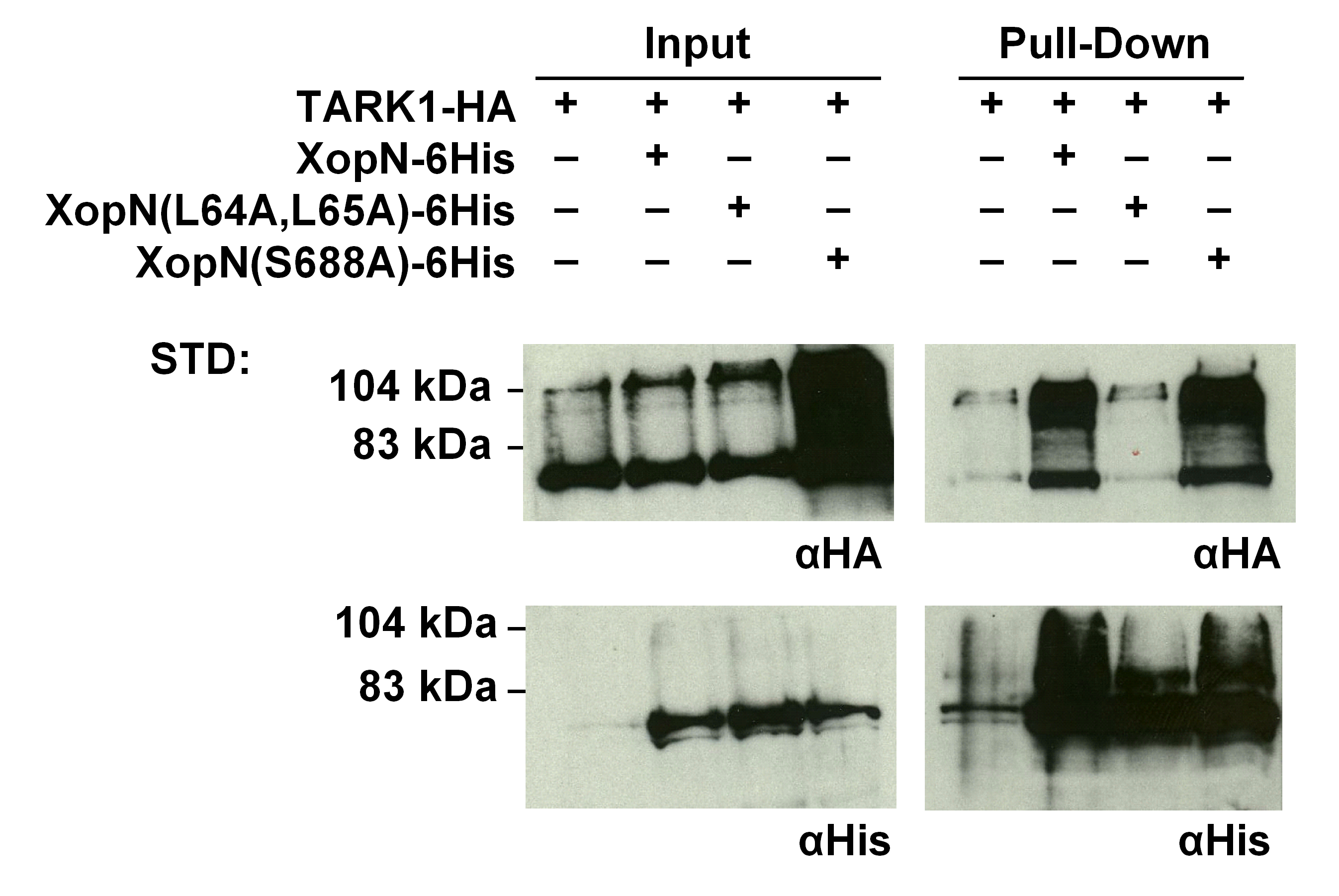

Supplement: Figure S8 — XopN(S688A)-6His interacts with TARK1-HA in pull-down assay. Pull-down analysis of TARK1-HA and XopN-6His, XopN(L64A,L65A)-6His, or XopN(S688A)-6His transiently over-expressed in N. benthamiana leaves using Agrobacteria. Leaves were hand-infiltrated with a 6×10^8^ CFU/mL suspension of A. tumefaciens expressing TARK1-HA or co-expressing TARK1-HA and XopN-6His, XopN(L64A,L65A)-6His, or XopN(S688A)-6His. After 48 hours, protein was extracted, purified by Ni+ affinity chromatography, and then analyzed by protein gel blot analysis using anti-His and anti-HA sera. Expected protein MW: TARK1-HA = 67.9 kDa; XopN-6His, XopN(L64A,L65A)-6His, XopN(S688A)-6His = 78.7 kDa. +, protein expressed; −, vector control. STD, molecular weight standard shown in kDa. (TIF) [file ppat.1002768.s008.tif]

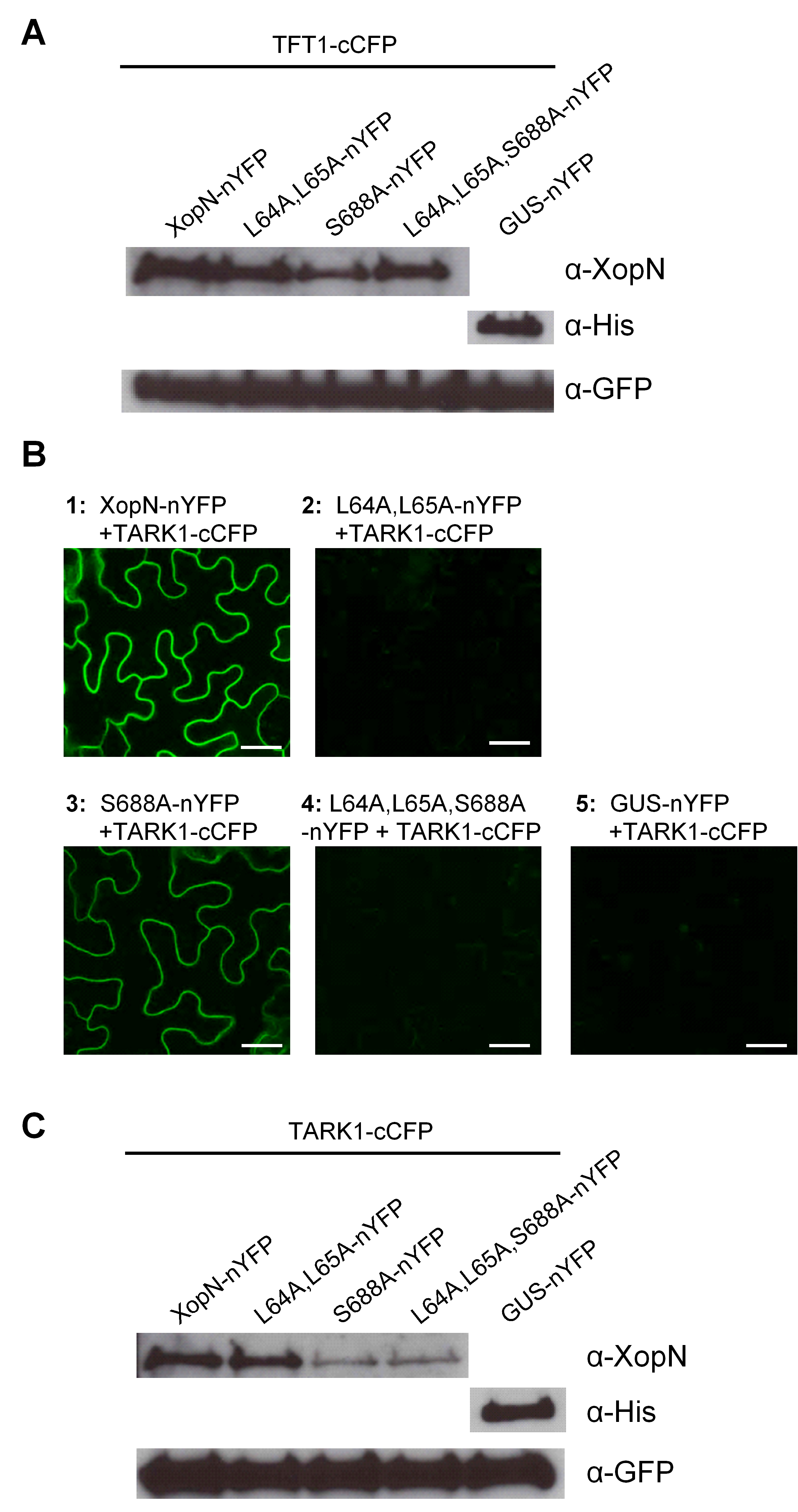

Supplement: Figure S9 — Protein gel blot analysis and confocal microscopy for BiFC analyses. (A) Protein gel blot analysis of the BiFC assay monitoring XopN/TFT1 interactions shown in Figure 8B . Anti-XopN, anti-His and anti-GFP sera were used. (B) BiFC assay of XopN/TARK1 interactions in N. benthamiana leaves. Leaves were hand-infiltrated with a 8×10^8^ CFU/mL total suspension of two A. tumefaciens strains expressing different fusion proteins (i.e. XopN-nYFP+TARK1-cCFP; L64A,L65A-nYFP+TARK1-cCFP; S688A-nYFP+TARK1-cCFP; L64A,L65A,S688A+TARK1-cCFP; or negative control GUS-nYFP+TARK1-cCFP) and then visualized by confocal microscopy at 48 HPI at 63X. White bar = 25 µm. (C) Protein gel blot analysis of the BiFC assay in (B) above. Anti-XopN, anti-His and anti-GFP sera were used. (TIF) [file ppat.1002768.s009.tif]

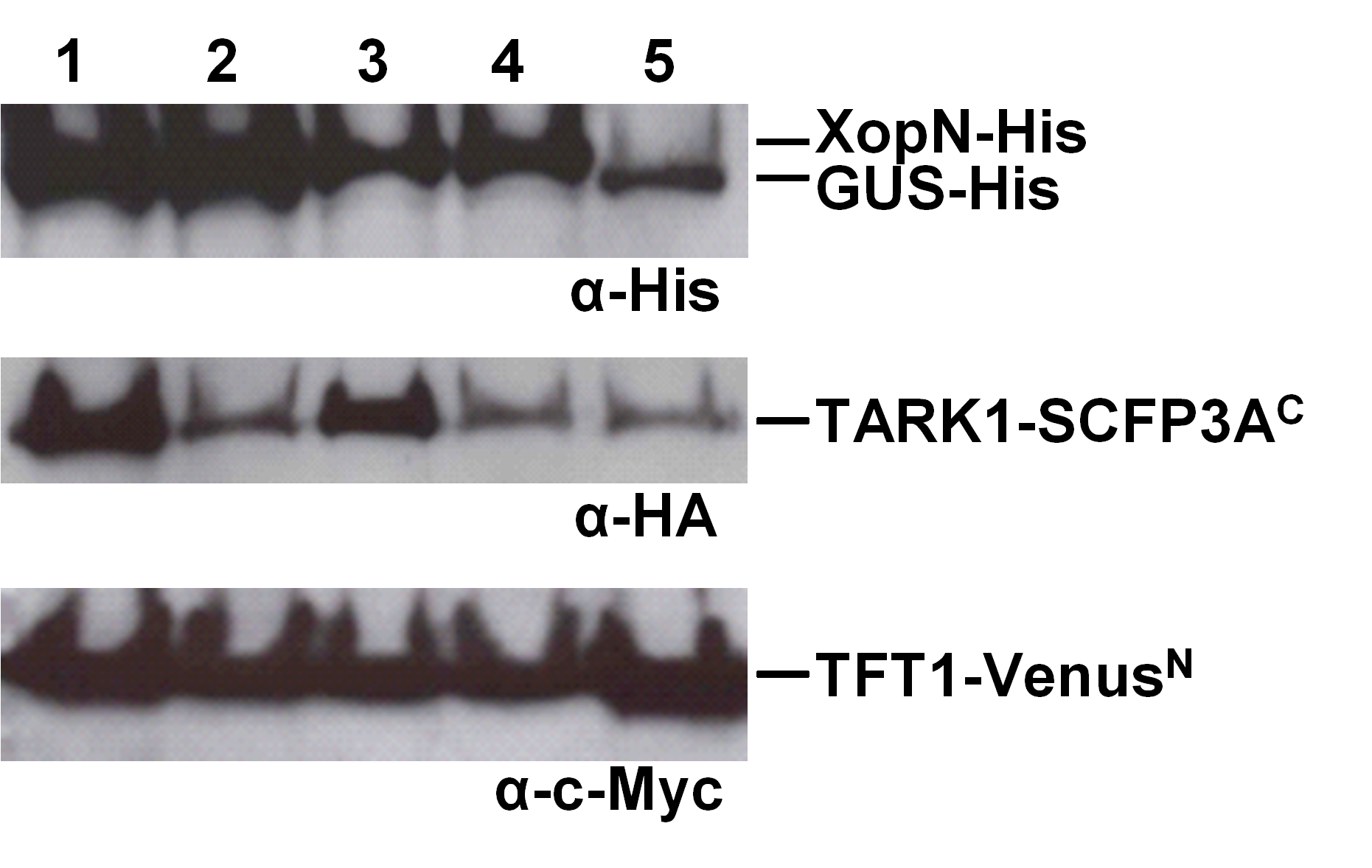

Supplement: Figure S10 — Protein gel blot analysis for TARK1/TFT1 BiFC assays shown in Figure 9A-E . Proteins were isolated from infected N. benthamiana leaves at 48 HPI and then analyzed by gel blot analysis using anti-His, anti-HA, and anti-c-Myc sera. Lane 1: XopN-6His+TARK1-SCFP3Ac+TFT1-VenusN; Lane 2: XopN-(L64A,L65A)-6His+TARK1-SCFP3Ac+TFT1-VenusN; Lane 3: XopN-(S688A)-6His+TARK1-SCFP3Ac+TFT1-VenusN; Lane 4: XopN-(L64A,L65A,S688A)-6His+TARK1-SCFP3Ac+TFT1-VenusN; and Lane 5: GUS-6His+TARK1-SCFP3Ac+TFT1-VenusN. VenusN domain has the c-Myc epitope. SCFP3Ac domain has the HA epitope. (TIF) [file ppat.1002768.s010.tif]
