# Supplementary material for: Tomato TFT1 Is Required for PAMP-Triggered Immunity and Mutations that Prevent T3S Effector XopN from Binding to TFT1 Attenuate Xanthomonas Virulence
Source: PLoS Pathog. 2012 Jun 14;8(6):e1002768. doi: 10.1371/journal.ppat.1002768 (PMC3375313; doi:10.1371/journal.ppat.1002768)
Supplement: Table S1 — List of primers used in this study. (DOC) [file ppat.1002768.s011.doc]

**Table S**1: List of primers used in this study.

| **Description** | **Primer Name; Sequence (5’-3’)** |
| --- | --- |
| **Primers to construct XopN deletion and site-directed mutants cloned into pENTR/D/TOPO vectors** | |
| N | BS1; CACCATGAAGTCATCCGCATCCGT  BS4; TCACATCGCGCCTGTCTTGCTGAG |
| C | BS3; CACCAAGACAGGCGCGATGCGT  BS2; TCACACCAGGTGCTTGGAGTGTT |
| M3 | BS5; CACCGGCAGCTTCCTCACCAAT  BS2; TCACACCAGGTGCTTGGAGTGTT |
| M4 | BS6; CACCCAATCGGCGGTTGTCAGC  BS2; TCACACCAGGTGCTTGGAGTGTT |
| M5 | BS1; CACCATGAAGTCATCCGCATCCGT  BS7; TCACTCGCGACGGTTGTTCTTG |
| M6 | BS1; CACCATGAAGTCATCCGCATCCGT  BS8; TCACGACAGCACGGACTTCTC |
| S688A | BS32; CGGGAACATGTTGCGGCCCCGAGTAGC  BS33; GCCGGGAACATGTTGCGGCCGCGAGTAGC |
| S688D | JG684; CGGGAACATGTTGATGCCCCGAGTAGC  JG685; GCTACTCGGGGCATCAACATGTTCCCG |
| S688E | JG686; CGGGAACATGTTGAGGCCCCGAGTAGC  JG687; GCTACTCGGGGCCTCAACATGTTCCCG |
| **Primers to construct XopN mutants cloned into pEZRK vectors** | |
| N | JG282; GGGATCCATATGAAGTCATCCGCATCCGTC  JG283; GGTCTAGATCAATGATGATGATGATGATGCATCGCGCCTGTCTTGCTGA |
| C | JG284; GGGATCCATATGAAGACAGGCGCGATGCG  JG285; GGTCTAGATCAATGATGATGATGATGATGCACCAGGTGCTTGGAGTGTT |
| L64A,L65A,S688A | JG282; GGGATCCATATGAAGTCATCCGCATCCGTC  JG285; GGTCTAGATCAATGATGATGATGATGATGCACCAGGTGCTTGGAGTGTT |
| M5 | JG282; GGGATCCATATGAAGTCATCCGCATCCGTC  JG656; GGTCTAGATCAATGATGATGATGATGATGCTCGCGACGGTTGTTCTTG |
| **Primers to construct XopN mutants cloned into pVSP61 vectors for Xcv85-10 ΔxopN complementation** | |
| M5 | JR170; GAGGTATCTCACAATGAAAGTGCGAGCTGC  KT34; GGGATCCTCAAGCGTAATCTGGAACGTCATATGGATACTCGCGACGGTTGTTCTTG |
| Forward and Reverse primers to amplify various XopN mutants | JR170; GAGGTATCTCACAATGAAAGTGCGAGCTGC  JR227; GGATCCTCAAGCGTAATCTGGAACGTCATATGGATACACCAGGTGCTTGGAG  TGTTTGCC |
| **Primers used for real-time RT-PCR** | |
| Actin | JG234; GAGCGTGGTTACTCGTTCA  JG136; CTAATATCCACGTCACATTTCAT |
| PR-1b1 | JG242; CTGGTGCTGGGGAGAATC  JG243; GTCCGATCCAGTTGCCTACA |
| TFT1 | JG660; GCCTCGTCCATCTGCTCCTG  JG661; GAATGCATCAGAAAAAGCATGCAG |
| TFT3 | JG668; GGAAGATCCCAAACCTGAAGAAAA  JG669; CTTAATGGAAAATCATAATACTAGCAAGG |
| TFT6 | JG670; GCTACACCTAAACCAGATGATAATGAAT  JG671; GATCTAGAACTAACAAAGAGTTGAATCCAG |
| PTI5 | CA305; ATTCGCGATTCGGCTAGACATGGT  CA306; AGTAGTGCCTTAGCACCTCGCATT |
| GRAS4 | CA297; CCGTCCTGATTTATTCATCCAT  CA298; TCGTGTGACGAAAAATGGAGT |
| WRKY28 | CA301; ACAGATGCAGCTACCTCATCCTCA  CA302; GTGCTCAAAGCCTCATGGTTCTTG |
| LRR22 | CA293; AAGATTGGAGGTTGCCATTGGAGC  CA294; ATCGCGATGAATGATCGGTGGAGT |
